# Dysbiosis From a Microbial and Host Perspective Relative to Oral Health and Disease

**DOI:** 10.3389/fmicb.2021.617485

**Published:** 2021-03-05

**Authors:** Carla Cugini, Narayanan Ramasubbu, Vincent K. Tsiagbe, Daniel H. Fine

**Affiliations:** Department of Oral Biology, Rutgers School of Dental Medicine, Newark, NJ, United States

**Keywords:** oral microbiology, oral immunology, commensal, pathobiont, metaproteomics, metabolomics, periodontitis, caries

## Abstract

The significance of microbiology and immunology with regard to caries and periodontal disease gained substantial clinical or research consideration in the mid 1960's. This enhanced emphasis related to several simple but elegant experiments illustrating the relevance of bacteria to oral infections. Since that point, the understanding of oral diseases has become increasingly sophisticated and many of the original hypotheses related to disease causality have either been abandoned or amplified. The COVID pandemic has reminded us of the importance of history relative to infectious diseases and in the words of Churchill “those who fail to learn from history are condemned to repeat it.” This review is designed to present an overview of broad general directions of research over the last 60 years in oral microbiology and immunology, reviewing significant contributions, indicating emerging foci of interest, and proposing future directions based on technical advances and new understandings. Our goal is to review this rich history (standard microbiology and immunology) and point to potential directions in the future (omics) that can lead to a better understanding of disease. Over the years, research scientists have moved from a position of downplaying the role of bacteria in oral disease to one implicating bacteria as true pathogens that cause disease. More recently it has been proposed that bacteria form the ecological first line of defense against “foreign” invaders and also serve to train the immune system as an acquired host defensive stimulus. While early immunological research was focused on immunological exposure as a modulator of disease, the “hygiene hypothesis,” and now the “old friends hypothesis” suggest that the immune response could be trained by bacteria for long-term health. Advanced “omics” technologies are currently being used to address changes that occur in the host and the microbiome in oral disease. The “omics” methodologies have shaped the detection of quantifiable biomarkers to define human physiology and pathologies. In summary, this review will emphasize the role that commensals and pathobionts play in their interaction with the immune status of the host, with a prediction that current “omic” technologies will allow researchers to better understand disease in the future.

## The Role of Microorganisms in Disease

### Introduction

In the context of homeostatic balance between the host and its microbial content, it has been proposed that the commensal microbiota plays a critical role in maintaining health (Lloyd-Price et al., 2016). Disruption of this homeostatic balance, is known as dysbiosis, defined as perturbations in the composition of commensal communities relative to that found in health (Petersen and Round, 2014; Lloyd-Price et al., 2016; Hooks and O'Malley, 2017). Dysbiosis can occur as a result of a change in the microbiota or the host's ability to respond to its microbiota. This delicate balance between homeostasis and dysbiosis is in part now seen as the early training of local and systemic immune regulation (both innate and acquired regulators) (Alm et al., 2002; Rook et al., 2013; Cox et al., 2014). Conceptually, this concept can be illustrated in germ-free animals where a lack of exposure to a typical commensal microbiota leads to an immature/untrained immune system (Falk et al., 1998; Macpherson and Harris, 2004; Round and Mazmanian, 2009; Sommer and Backhed, 2013; Kennedy et al., 2018). In contrast, early exposure and immune training as seen in immune competent humans, has been termed the “old friends” hypothesis, which describes the manner by which the host responds to microbial challenges (Rook, 2010; Cox et al., 2014; Rook et al., 2014). The disturbance of training of the immune system, also seen in humans, has been illustrated by challenging the infant commensal microbiota with antibiotics, which can disrupt this homeostatic balance (Dominguez-Bello et al., 2019). Moreover, antibiotics given to infant mice can change their gut (and likely oral) microbiota, suppress the normal (eubiotic) commensal microbiota, and add weight (obesity) and height to these antibiotic-treated mice (Cox et al., 2014; Lamont et al., 2018).

Further substantiation of this need for training and balance has been shown by removal of intestinal microbial contents from non-antibiotic treated mice followed by transplantation of the intestinal microbiota from antibiotic treated infant mice as compared to non-antibiotic treated mice (Cox et al., 2014; Ellekilde et al., 2014). When challenged, these two transplanted populations were shown to produce distinctive responses in the mice receiving the transplants. The newborn mice receiving the transplants from antibiotic treated mice produce heavier and larger mice as compared to those mice receiving the “normal” non-antibiotic manipulated microbiota (Cox et al., 2014). In experiments by Ellekilde et al. (2014) the ultimate goal was to develop a system designed to circumvent the need for germ free mice in order to document microbiome development and its effect on the host. Colonization of transplanted mice was assessed comparing donors from either lean or obese mice. The transplantation effect, in these experiments, although stable for only 6 weeks, was sufficient to train the immune system and allow for the study of pathogenesis in murine models of disease.

The importance of this “early” commensal gut microbiota in brain development has also been described in both studies with mice and humans (Lu et al., 2018; Lu and Claud, 2019). Proper neurological development appears to be intimately tied to maintenance of a healthy microbiome, and alternations or dysbiosis appear to be linked to schizophrenia, autism spectrum disorders, and hyperactivity disorders (Hsiao et al., 2013; Kong et al., 2019; Lu and Claud, 2019). Gnotobiotic mice humanized with transplanted early fecal microbiota from preterm infants with either good or poor growth, and mouse brain, liver, fecal, and serum samples were obtained to analyze histology, protein, fatty acid, and RNA expression levels in these transplanted mice (Lu et al., 2018). Mice that were colonized by poor-growth microbes showed decreased levels of markers of early development in the brain, and delayed oligodendrocyte development and myelination, indicating a delay in neuronal development. Furthermore, in the poor-growth mice, neurotransmitter levels were altered and animals developed neuroinflammation. There was a subsequent change in the short chain fatty acids from the gut microbiota. This study demonstrated the profound effect the colonizing microbiome had on early brain development, validating many of the theories and prior data linking the gut-brain axis in neuronal development (Lu et al., 2018).

### Are Microbes Alone Responsible for Health or Disease?

This “old friends” mechanism coupled with the important work of Casadevall and Pirofski clearly point out that it is inadequate and misleading to define disease based solely the virulence capability of specific microbes and the so-called “pathogenic microbiota” (Casadevall and Pirofski, 2015, 2018). Concomitantly, health should be defined in terms of the influence of the commensal microbiota (“old friends”) on its host response capabilities. Therefore, the host as well as the microbiota should be included when assessing health or disease (Dominguez-Bello et al., 2019). From the perspective of disease, this is best explained in the Damage/Response Framework that demands that we define disease in the context of the host (Casadevall and Pirofski, 2003; Pirofski and Casadevall, 2008). Thus, typically harmless bacteria can become opportunistic or pathogenic, and are capable of inducing disease when introduced into an organ system that is not its normal ecological niche especially in immunologically compromised individuals (Casadevall and Pirofski, 1999). From the perspective of health, one should consider the importance of the commensal microbiota and how its absence or alteration can undermine immune surveillance and influence growth, development, and resistance to disease (Dominguez-Bello et al., 2019). As mentioned in the Damage/Response Framework a defective immune system can have severe clinical implications in disease. This was illustrated initially in the case of HIV/AIDS (Casadevall and Pirofski, 1999). In the Framework proposed in this conceptualization of disease, microbes such as the opportunistic pathogen *Cryptococcus* sp., or commensals like *Candida* sp. or *Staphylococcus* sp., that are ordinarily controlled by immune competence, now become the cause of morbidity and mortality. Thus, in the Damage/Response Framework generally “harmless” bacteria, fungi, and viruses now run rampant and cause fulminating infections that cannot be controlled at either the local of systemic level (Casadevall and Pirofski, 1999).

### How Does the Commensal Microbiota Evolve?

Higher order organisms acquire their microbiomes from their immediate environment upon birth initially through oral feeding. Mammals are known to be colonized initially by organisms present in the birth canal and passively from primary caregivers during rearing (Berkowitz and Jones, 1985; Lamell et al., 2000; Mueller et al., 2015). Barring any disruption from outside influences (i.e., antibiotics while *in utero* or early in the colonization process) or imbalances in the maternal microbiome, offspring will acquire their healthy commensal microbiome early in their development (Cho and Blaser, 2012). Commensals, our “old friends,” play an important role in homeostasis, disease control through competition (i.e., for nutrients) and exclusion (i.e., inhibitory compound production) of pathogens, maintaining health at local sites through adequate colonization rates, metabolic activity, and immune training (Relman, 2012; Abt and Pamer, 2014). The importance of the commensal microbiota in the protection from potentially pathogenic species is well-illustrated in the case of commensal *Neisseria* species of the oro-nasopharynx (Dorey et al., 2019). *Neisseria*, which are regarded as benign common colonizers of the mouth and nasal cavities are generally able to manage pathogenic or pathobiotic *Neisseria* species. For instance, indigenous *Neisseria* can kill the potential pathogenic *Neisseria gonorrhoeae*, or likely outcompete the pathobiont *Neisseria meningitidis* (Pandey et al., 2018; Dorey et al., 2019; Kim et al., 2019).

It has now become clear that there is a selective process that determines the colonizing order and distribution of microorganisms that gather on epithelial and hard surfaces forming the microbiome of humans and other mammals (Shafquat et al., 2014; Lloyd-Price et al., 2016). Previous theories suggested that microorganisms were ubiquitous, completely surrounding and inhabiting us based on selection preferences that were poorly defined. Concisely put, the prevailing theory was that “everything is everywhere” (O'Malley, 2007). Overtime this concept has evolved into a more precise definition of ecological selection (Costello et al., 2012; Foster et al., 2017). Currently, the local environment still forms a key element in the selection process. However, contemporary concepts now indicate that both the niche microbiota and the host response to that microbiota serve as a filter for microbial selection and the successional development of specific habitats (Human Microbiome Project, 2012; Foster et al., 2017).

## Studies Pointing to the Importance of Microbial and Host Involvement in Infectious Diseases

### Interest in the Prominence of Dysbiotic Microbial Communities

In a natural state, most niches are filled by a climax community that has unique physiological and/or metabolic demands that therefore can restrict invasion or colonization of non-niche or transient species. However, successful disruption of this community (“patch or domain”) will permit shifts in the established inhabitants that can lead to dysbiotic behavior and potentially pathogenic communities (Relman, 2012). Microbial species that are original occupants of a specific niche are functionally fit for that ecological niche (Polechová and Storch, 2019). Interconnecting food chains allow for a multitude of physiological functions that permit microbial diversity in a “climax community” (Jorth et al., 2014). Dispersal, local diversification, environmental selection, or, ecological drift can allow for subtle or not so subtle shifts in the climax community (Costello et al., 2012).

#### Chemical and/or Physical Causes of Ecological Disruption

To illustrate this more specifically either chemical or physical disruptions can lead to microbial community imbalances or dysbiosis. Chemically induced environmental dysbiosis can be seen in the overuse of antibiotics, which creates an ecological catastrophe, particular within the gastrointestinal (GI) microbiota (Relman, 2012; Dominguez-Bello et al., 2019; Cullen et al., 2020).

In the past, Clindamycin had the highest association with GI disturbance and serious consequences among antibiotics studied (Sullivan et al., 2001; Brown et al., 2013). In addition, older patients appeared to be more susceptible to the effect of Clindamycin in its ability to disrupt intestinal equilibrium (Loo et al., 2011). The overall consequence was the overgrowth of *Clostridium difficile*, and suppression of microbes that could counter its effect and thus disrupt homeostasis. In a mouse model a single dose of clindamycin was able to render the animals susceptible to *C. difficile*-induced colitis (Buffie et al., 2012). This is just one example of chemically induced environmentally initiated dysbiosis resulting from the misuse of antibiotics, which creates an ecological catastrophe.

Recent work addressing the impact of orally administered prophylactic antibiotics on the gut microbiota of hematology patients was observed (Willmann et al., 2019). Patients were immunocompromised due to their malignancies and were administered either ciprofloxacin or co-trimoxazole daily depending on the study site. Baseline laboratory tests were performed to assess liver function and markers of infection. Study participants gave stool samples prior to antibiotic treatment, days 1 and 3 post-initiation of treatment, and in the final antibiotic dosage period, which had a 6-day median time period. The samples were evaluated by shotgun sequencing, whereby in addition to speciation, the resistome and plasmidome could be analyzed. In both groups there was an observed decline in the Shannon diversity at a phylum level over the course of the treatment, which showed specific microbial species depending on the antibiotic. Antibiotic resistance genes increased over the course of the study, but the specific genes were dependent on the antibiotic. Interestingly, the patient's laboratory findings also correlated with alternations in the microbiota, pointing to the role the host has in shaping the gut microbiome.

Another example of the importance of the normal commensal microbiota and a consequence of its disruption can be shown by the homeostatic microbial imbalance and immune disruption caused by overuse of antibiotics (Dethlefsen et al., 2008; Willing et al., 2011). Recent studies observed the change in the microbiota and an alteration of cytokine release in 3-week-old female C57BL/6 mice (Sun et al., 2019). Mice were given sterile water, enrofloxacin, vancomycin, or polymyxin B for 3 weeks. Their colons were removed and analyzed for histology, cytokine gene expression profiles, 16S rRNA sequencing, and metabolome analyses. Histology was largely unremarkable between samples. However, all three antibiotic treatments significantly up-regulated the gene expression of pro-inflammatory (*IFN-*γ, *TNF-*α, *IL-1*β, and *IL-6*) and anti-inflammatory cytokines (*IL-4, IL-17, IL-23*, and *IL-10*), but varied in fold-change depending on the treatment. Both vancomycin and enrofloxacin decreased the species richness and diversity indices of the colon microbiota. Further changes in fatty acid and amino acid metabolites were seen, which correlated with the presence of select microbial taxa.

In light of these illustrations we suggest that the oral cavity is no different from other ecological systems in the sense that it will build its microbial climax community based on its environmental components.

### The Oral Cavity and Microbiome Analysis

Our oral microbiota is acquired at birth and over time from our primary care givers (Berkowitz and Jones, 1985; Lamell et al., 2000). The microbiome of the oral cavity has been studied since the 1960 where researchers began to appreciate that the supra and subgingival microbiota was composed of a complex consortium (Socransky et al., 1987; Fine, 2006). From the 1990s on there was a push to define all microorganisms present, and one could argue that the oral cavity was one of the original human-associated microbiomes to be characterized (Socransky and Manganiello, 1971; Socransky and Haffajee, 1991, 1994; Socransky et al., 1998). There was an appreciation for the fact that the oral cavity consisted of a consortium that is associated with disease, complicating the way in which infections were described. This new appreciation pushed for a revision of Koch's postulates, reformulated by oral microbiologists as Socransky revision of Koch's postulates (Socransky, 1979; Socransky and Haffajee, 1991).

Distinctive oral sites appear to be packed with commensals and these commensal can change from birth to senescence based on environmental changes in salivary flow and content, tissue rigor, hormonal conditions, diet, etc. Immediately after tooth brushing a succession of events occur on a tooth surface (Socransky and Manganiello, 1971). Pioneer colonizers, consisting mostly of Gram-positive bacteria, collect on the enamel surface in parallel arrays extending from the tooth surface (Kolenbrander, 2000; Kolenbrander et al., 2002, 2006; Li et al., 2004; Hojo et al., 2009; Esberg et al., 2020). These pioneers are succeeded by secondary and tertiary species all of which are commensal members of the oral microbiota (“normal” inhabitants of the oral cavity). The pioneer species, the hardiest of the oral microbial species, attach avidly to salivary-coated enamel surfaces (salivary pellicle) and form a resistant/adherent band of microbes. Astonishingly, these microbes were first identified in 1678 by Antonie van Leeuwenhoek as tiny “animalcules” (James, 1994; Lane, 2015). The pioneer primary colonizers followed by secondary and tertiary colonizers to a large extent make up our protective commensal microbiota, while also harboring potential pathobionts. Pathobionts are natural members of the human microbiota that have pathogenic potential under certain conditions (Mazmanian et al., 2008; Cugini et al., 2013).

Overall, there have been over 1,000 species identified that have the potential to make up the oral microbiome and we suspect that 70–100 species are present in any one individual (Dewhirst et al., 2010; Park et al., 2015; Xu et al., 2015). These numbers consist of the high abundance commensals as well as the lower abundance pathobionts and microbes of unknown function or role in disease. For disease either a physical assault or chemical biofilm induced-irritation, can result in a pathogenic biofilm that is characterized by out-growth of select species, which can give rise to many of the infections that arise in the oral cavity.

### The Oral Microbiome: A Brief Historical Overview

Given that the oral microbes have been studied since the times of von Leuwenhoek, many of the early microbiologists were keen to study these bacteria and devised ways to study them *ex vivo*. One of the first intensive studies of oral microbes involved in disease was performed in the laboratories of Dr. Robert Koch by W. D. Miller, a visiting dentist from the United States. Miller, in a series of detailed experiments, clearly demonstrated the preference of oral microbes for carbohydrates and their relationship to acid production and caries (Miller, 1890). However, the main caries-culprit was first identified as *Streptococcus mutans* by Clarke, an English microbiologist in 1924. His description went largely un-noticed and therefore the relationship between these acid-loving, acid-producing oral microbes and caries was not totally accepted until the elegant experiments of Paul Keyes in 1964 (Clarke, 1924; Englander and Keyes, 1964). Keyes who was working on the effect of diet on caries made the serendipitous discovery that golden hamsters had caries while albino hamsters had none (Fitzgerald and Keyes, 1963; Englander and Keyes, 1964). After isolation of the microbes from the mouth of golden hamsters, inoculating pure cultures into the caries-free albino hamster, he showed how caries evolved. Keyes then did a series of experiments to show this microbe/host relationship in many well-designed experiments of experiment of which one in particular is worth highlighting. In this experiment, Keyes took albino pups delivered by Cesarean section in a germ-free chamber and housed these pups with golden hamster surrogate mothers, demonstrating that the caries-producing microbes could be passed from golden hamster mom to the albino pups and that the pups now showed carious lesions (Fitzgerald and Keyes, 1960; Keyes and Fitzgerald, 1962). In contrast, he took golden hamster pups and placed them in the cage with the albino mom. He showed that the golden hamster pups did not get caries and that the caries-producing organism could not be recovered from either the mom or the pups. These elegant experiments focused attention on *Streptococcus mutans* and led to many experiments attempting to show that caries was an infection caused by a specific microbes.

The microbiota associated with periodontal disease also served as an area of research. In the earlier studies, Rosebury and colleagues in the 1930's and others (e.g., Kritchevsky and Seguin) made efforts to isolate microbes from periodontal pockets and showed how they provoked infections in a guinea pig groin model (Kritchevsky and Seguin, 1918; Rosebury et al., 1929, 1934). They concluded that a mixture of microbes was required and no single bacteria could provoke infection, but that a pathogenic quartet of microbes appeared to result in disease. These studies were challenged by Rosebury's graduate student J. B. MacDonald who pointed to *Bacteroides melaninogenicus* as the prominent pathogen related to the cause of periodontal disease (Macdonald et al., 1956). These controversies led to a series of alternative hypotheses developed by Dr. Walter Loesche, a former student of the MacDonald group (Table 1; Loesche, 1976), He introduced two hypotheses: (1) The Non-Specific Plaque Hypothesis (NSPH), and (2) Specific Plaque Hypothesis (SPH). The NSPH stated that disease was unrelated to specific microbes but rather was related to an accumulation of products derived from masses of microbes. In contrast, the SPH stated that a particular microbe provoked disease. These alternative hypotheses provided a window into the way in which infectious periodontal diseases could evolve. In time, these hypotheses evolved into the Ecological Plaque Hypothesis which states that the environment dictates the microbiota and must therefore be considered in terms of disease (Marsh, 1994). This theory is closest to how we understand microbial induced dental diseases today and in many ways illustrates a hypothesis that parallels the Damage/Response Framework (Casadevall and Pirofski, 1999).

**Table 1:** Criteria for defining disease causation.

**Criteria for defining disease causation**	**Date**	**Novelty**	**Focus**	**References**
Koch's postulates	1893	Initial efforts to identify disease causation	Microbes	Koch, 1893
Loesche's criteria	1976	Efforts to examine microbial causes of dental diseases	Microbes	Loesche, 1976
Socransky modification of Koch's	1991	Efforts to include host in Koch's postulates	Microbes and Host	Socransky and Haffajee, 1991
Marsh ecological criteria	1994	Efforts to establish ecological influences on dental diseases	Microbes with ecological consideration	Marsh, 1994
Casadevall and Piroski Damage/Response	1999	Effort to understand host's participation in infectious diseases	Microbes in relationship to Host	Casadevall and Pirofski, 1999

## Examples of Commensals and Pathobionts in Oral Disease

**Figure 1:**
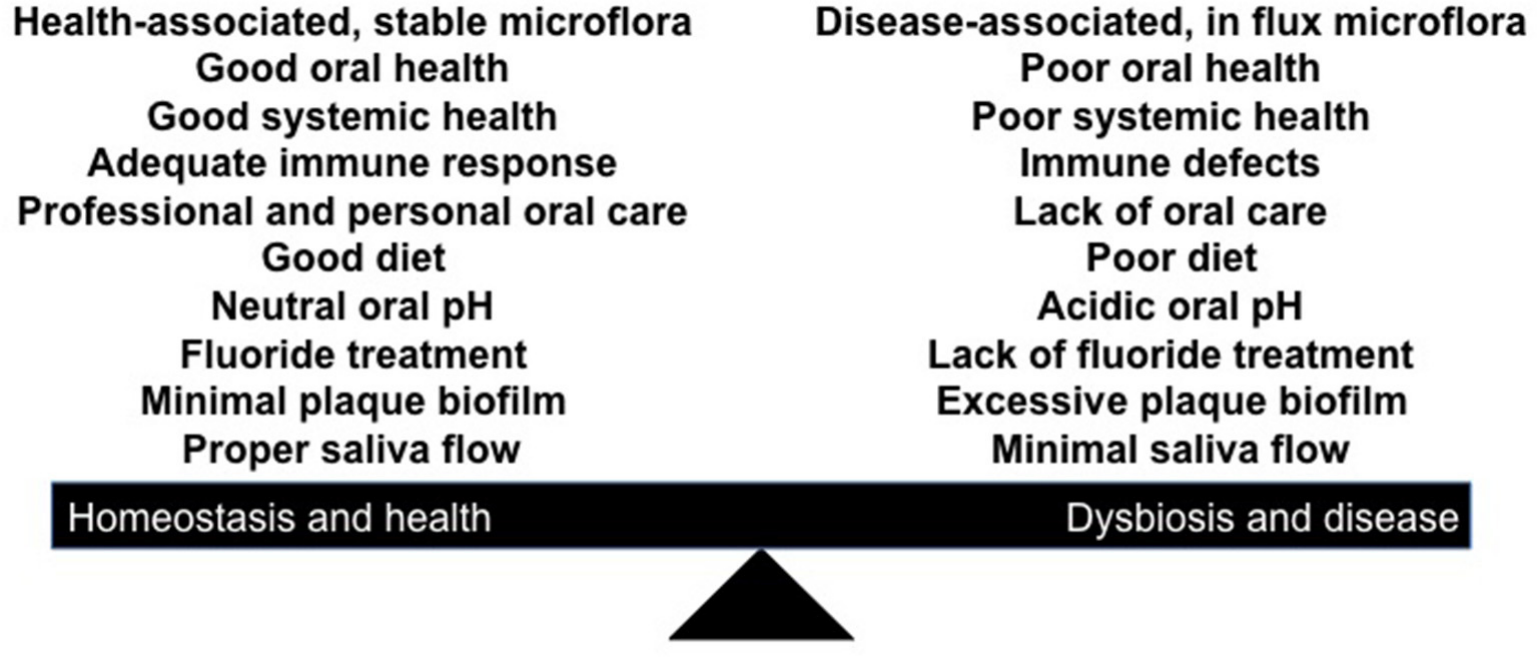
Illustration of a shift in the homeostatic balance that favors dysbiosis in favor of disease. Influences on the right side that favor disease can include; physical, chemical, or biological influences that can disrupt the balance. Factors such as iatrogenic dentistry, nutrition (excess carbohydrates; type of diet), lack of salivary flow, changes in local pH, defects in enamel mineralization, inadequate immune responsiveness, leukocyte adhesion defects, etc. all can have a profound influence on dental disease at the local level.

For dental disease to occur we propose that either a physical assault, chemical biofilm induced-irritation, and/or biological induced changes can result in a “pathogenic or disease promoting biofilm” (Figure 1). This putative “pathogenic” biofilm is characterized by outgrowth of select species, which we suggest can give rise to the most prevalent infections (periodontitis and caries) that arise in the oral cavity.

### Periodontal Disease

In a microbiome at homeostasis there are delicate interspecies interactions driven by the maintenance of intricate physical and metabolic associations. These associations exert control over the host innate immune defenses in their effort to detoxify the environment. In periodontal disease there is a breakdown of this homeostasis that is characterized by the formation of a dysbiotic biofilm, plaque, and outgrowth of key pathobionts in the microbiome, which leads to host tissue destruction and ultimately, formation of periodontal pockets and bone loss. These chronic inflammatory diseases begin as reversible gingival inflammation (gingivitis), and if not managed, leads to advanced periodontal disease (Moore and Moore, 1994). Periodontitis results in increased bone resorption around the tooth and root area, which leads to eventual tooth loss. In chronic periodontal disease the host's oral microbiome shifts from a predominately Gram-positive healthy plaque biofilm to a pathogenic and primarily anaerobic dysbiotic consortium (Marsh and Zaura, 2017). The initiating events are poorly understood but it is likely a physical irritation that causes initial changes in the local environment, which allows for nutrient sources and an alteration in the local innate immune response. The Gram-negative pathobionts, while previously existing as low-abundance species begin to proliferate (Haffajee et al., 2008; Dewhirst et al., 2010; Uzel et al., 2011; Chen et al., 2018; Lamont et al., 2018; Curtis et al., 2020).

#### Physical Assaults That Can Result in Dysbiosis

**Figure 2:**
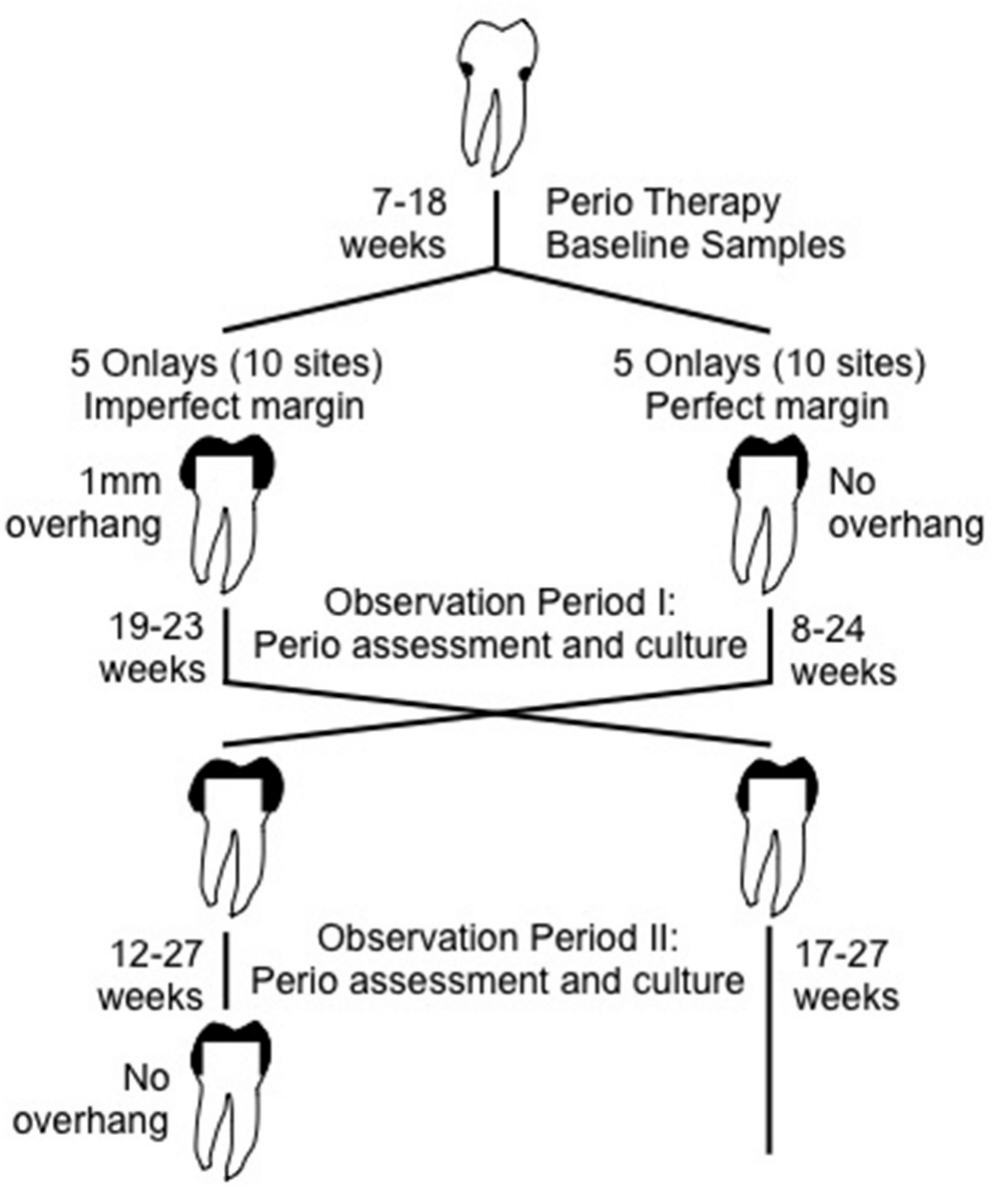
Illustration of experiment showing how an overhanging dental restoration can influence homeostatic balance by influences on tissue integrity, inflammatory responsiveness etc. As such a physical change in the local environment that can produce a local inflammatory response that can lead to increased presence of red blood cells and cause an ecological shift in the microbial balance influencing the overgrowth of an anaerobic, heme dependent subgingival microbiota. Adapted from Lang et al. (1983).

Periodontal wounds can be caused by overhanging restorations, which can result in ulcerations of soft tissue leading to inflammatory changes in the underlying tissues. Early studies by Waerhaug (1956) clearly showed that roughly surfaced dental restorations placed below the gingival margin created histological changes in the underlying gingival tissue. In a dramatic illustration of this iatrogenic effect, one study took 9 dental students who needed mesio-distal inlay restorations (Lang et al., 1983). The restorations were place in the student's mouths and they were followed for tissue changes, inflammation, and microbiology over time (Figure 2). In one group, the restoration was perfectly fitted so that no tissue irritation would occur below the gum margin. The other group had a restoration that was designed to have a poorly constructed restoration with an “overhanging” metal margin that served to irritate the underlying tissue. The restoration was kept in place for 19–23 weeks, during which time the clinical condition was recorded as was bleeding on palpation every 2–3 weeks. The area below the gum was also sampled for predominant types of bacteria, which were determined by culture analysis of anaerobic bacteria. The restoration was then removed and redesigned such that the side that was perfectly fitted now had the “overhang” while the other side now had a well-fitted restoration, and the clinical and microbiological observations were continued as described. Bleeding on probing always was preceded by the presence of black-pigmented “Bacteroides” types of microbes, now known as Porphyromonads, microbes associated with gingivitis and periodontitis. These microbes were also associated with the overhanging margins, which were directly related to a change in the clinical condition, which led to the emergence of the Bacteroides type microbes. These changes document the potential for iatrogenic factors (poor dentistry) as an initiator of gingivitis and periodontitis causing a periodontal wound. Clinically this can be seen as an altered gingival sulcus now called a periodontal pocket.

This physical change can result in an alteration in microbial succession as compared to what typically takes place in an unadulterated environment. These changes can lead to bleeding and a change in the nutritional contents of the wound environment. Inflammation provides a specific set of nutritional factors, while bleeding provides red blood cells, hemin, and fibrin, factors that are useful nutritional and signaling metabolites for Bacteroidetes, Spirochetes, and Porphyromonads (Page and Schroeder, 1976). In the transition from health to disease the pocket deepens, anaerobiosis increases, available carbon sources decrease, and inflammation and pH changes occur which induce temperature changes. These changes contribute to a fluctuating ecology in the context of the semi-delineated periodontal pocket environment (Loesche, 1993). When the intact delineated epithelial barrier is broached or ulcerated, oral bacteria can escape from the pocket and enter the blood stream (Fine et al., 1996). Here colonies from the subgingival biofilm invade the circulation. The first bacteria to appear in the circulation can aptly be termed pathobionts (or amphibionts), which now can invade a foreign territory, by moving through the blood stream, where they can settle on an irregular vascular niche. Now either single or multiple species that move away from their natural habitat course through the bloodstream and settle on damaged vessels or tissues (i.e., streptococci, Fusobacteria, *Aggregatibacter*, Bacteroidetes, porphyromonads, etc.).

#### Chemical Assaults That Can Result in Dysbiosis

A good example of the influence of antibiotics on the interference with natural microbiome homeostasis is illustrated by an experiment where specific classes of antibiotics were applied to tooth surfaces after thorough debridement (Loe et al., 1967). Since the sequence of events in early tooth related biofilm development was known it was possible to select antibiotics that would interfere with that progression. It was shown that streptococci in the oral cavity were known to be the first to interact with salivary-coated enamel surface immediately after debridement. After forming parallel arrays perpendicular to enamel the interstices between these parallel arrays was colonized by *Veillonella*, a Gram-negative facultative anaerobe. This was followed by a mixture of both Gram-positive and negative bacteria that formed a complex biofilm community in a 10-day period after abstinence from tooth brushing. Application of vancomycin, which inhibited Gram-positive bacteria, resulted in a thinner less dense biofilm; while application of polymyxin B, which inhibited Gram-negative bacteria, showed less of an overall effect, but the specific composition of the biofilm was significantly altered. The most dramatic effect was seen when tetracycline (a broad spectrum antibiotic) was painted onto cleaned tooth surfaces whereby an overall reduction in plaque biofilm was seen over this 5-day period of brushing abstention. This elegant but simple experiment demonstrated the potent effect of antibiotics on a change in the homeostatic balance in natural biofilm formation on tooth surfaces.

In clinical settings it has become apparent that a better understanding of antibiotics is imperative in the balance between health and disease (Sharma et al., 2019). Antibiotic sensitivity and resistance have to be taken into consideration (Marcinkiewicz et al., 2013). Effects of short-term antibiotic therapy have been questioned because the long-term clinical effect is not significantly superior to non-antibiotic therapy in efforts to reduce the pathogenic or dysbiotic microbiota (Hagenfeld et al., 2018).

#### Biological Assaults That Can Result in Dysbiosis

This example is seen mostly in what is thought to be genetically modified host response diseases, one example of which is Leukocyte Adhesion Deficiency (LAD). In LAD patients have a defect in leukocyte adhesion to endothelial cells resulting in defective transmigration of leukocytes into tissues (Hanna and Etzioni, 2012; Silva et al., 2019). Defects in CD18 expression result in a lowered B2 integrin expression on endothelial cells and leukocytes fail to migrate into the adjacent connective tissue. As a result, tissues are challenged by microbes that do not get removed, become infiltrated with these microbes, and patients succumb to progressive periodontal disease and tooth loss (Moutsopoulos et al., 2015). Recent evidence suggests that a dysregulated host response results in upregulation of IL-17 a bone modulating cytokine (Hajishengallis and Moutsopoulos, 2014, 2016). A less dramatic case of a biological assault can be seen in Localized Aggressive Periodontitis whereby a leukotoxin produced by *Aggregatibacter actinomycetemcomitans* can result in limited success for polymorphonuclear leukocytes (PMNs), lymphocytes and macrophages in the modulation of bacteria resulting in overgrowth of bacteria that could ordinarily remain under control. Thus host protective lymphocytes and leukocytes are affected at the local level producing an aggressive form of disease (Fine et al., 2013).

### Caries

A second example of the prominent influence of the host on local oral disease can be seen in a description of caries. Caries (commonly called cavities) occur as a result of demineralization of enamel due to acid end-products generated by sugar consumption of oral bacteria that reside in dental plaque biofilm that collects on the enamel surface (Takahashi and Nyvad, 2011; Mira et al., 2017). Ultimately, there are key low-level acidogenic and aciduric pathobionts that reside in the cavity and due to a combination of tenacious biofilm formation, insufficient host innate response (i.e., saliva is not sufficient), and environmental factors (i.e., diet) these key organisms are allowed to survive in an otherwise inhospitable environment to other commensals. As the previously described chemically initiated environmental dysbiosis, these acidogenic and aciduric bacteria cause a dysbiosis of the local microbiome where only the most acid tolerant survive (Takahashi and Nyvad, 2011). In a healthy microbiome, the pioneer colonizers, in large part streptococci, form one's protective commensal microbiota and attachment sites for subsequent colonizers. Moreover, these organisms, in concert with other species create mutually beneficial environments for the colonizers (Kreth et al., 2005; Kolenbrander et al., 2006; Jakubovics et al., 2008a,b; Treerat et al., 2020). In rare instances these Streptococci species are responsible for extra-oral second site infections, such as endocarditis or abscess, hearkening back to Casadevall and Pirofski and the need to understand both the nature of the microbe and the host.

Here again we wish to stress the importance of homeostasis or more specifically the disruption of microbial and host homeostasis, which we term dysbiosis. As in the case of periodontitis we will divide these dysbiotic mechanisms into three categories; Physical, chemical, and biological factors that can influence a shift away from homeostasis to dysbiosis.

#### Physical Changes That Can Result in Dysbiosis

A physical constraint that encourages dysbiosis in the oral cavity occurs during tooth formation. The biting surfaces of molar teeth, called the occlusal surfaces, consist of pits and fissures, which are grooves and depressions in the top surface of teeth. The depth and tortuous anatomy of these surfaces play a role in how they contribute to the caries process. A deep pit or fissure in an occlusal surface can contribute to the accumulation of bacteria and forceful the packing of bacteria into these deep crevasses. As a result of this impaction, the bacteria residing in the depth of these recesses can rest in a protected domain and metabolize carbohydrates to produce acid and demineralize these surfaces (Fine, 1995). This process is different than what happens in smooth surface decay of enamel where the microbes attach by means of protein adhesins found on the surface of the pioneer bacteria or to glycan receptors due to salivary binding to enamel surfaces. For occlusal decay bacteria such as *Lactobacillus* sp. that do not attach on their own, can establish a home in the occlusal pit without a need for adhesins. Here the bacteria can metabolize carbohydrates to reduce the biofilm pH to below 5.5 and thus produce decay (Fine et al., 1996). To counter-act this issue researchers have developed sealants made of thin plastic that fill in the occlusal pits and fissures to prevent the penetration of bacteria into these deep crevasses.

Another example of a physical change that can contribute to dysbiosis is in tooth root induced by exposure to bacteria due to gingival recession. The root is composed of a less mineralized cementum containing more irregular surfaces that becomes exposed due to a receding gum-line resulting from periodontal disease or trauma to the gingiva. Here we find an exposed cemental surface that has anomalies providing opportunities for colonization by bacteria, in particular those that would otherwise not colonize as they lack adhesive properties, to lodge in these areas and produce root surface decay (Takahashi and Nyvad, 2016). These conditions allow for bacteria to remove organic and inorganic material from root surfaces leading to physical changes that make the root more vulnerable to decay.

#### Chemical Changes That Result in Dysbiosis

Stephan showed that acid formation resulting from ingestion of carbohydrates resulted in a sharp drop in the biofilm pH immediately adjacent to the enamel surface (Stephan and Miller, 1943). It was shown that with repeated exposure to carbohydrates, members of the commensal microbiota that thrive at a low pH overgrew and these reputed causative microbes thrived at a low pH, which caused environmental selection that reduced diversification and changed microbial homeostasis (Marsh, 1994). Thus low pH favors the growth and survival of acid tolerant and acid producing microorganisms that thrive at a low pH, in this manner limiting diversification in this local patch or domain (Kianoush et al., 2014). Thus, carbohydrate-consuming species multiplied became profligate and produced lactic and formic acid as an end product of their metabolism (van der Hoeven and Franken, 1984; Duguid, 1985; Dashper and Reynolds, 1996). The patch or landscape in which the now low pH-favoring microbiota lives is typically influenced by salivary flow. By virtue of the buffering capacity of bicarbonate and other elements, the saliva buffers this sugar-induced pH decline returning the area to a neutral pH (Vila et al., 2019). However, in keeping with the Damage/Response Framework we now recognize that even in the case of infusion of sugar, that creates dramatic ecological changes favoring the outgrowth of acid tolerant/acid producing microbes, the microbiota is constrained by its environment, which is partially controlled overtime by salivary influences.

#### Biological Changes That Result in Dysbiosis

In caries as mentioned above, a critical element in the carious process is pH, and once the pH in a biofilm drops below 5.5 enamel demineralization begins (Dawes, 2003). The influence of saliva (a local Damage/Response regulator) on this process is critical due to its buffering capacity (Local Response), which if lacking can be catastrophic. This has been illustrated in several ways. In one case, in the absence of salivary flow, it is clearly shown that the pH drop will continue unaltered and disease or tissue damage will occur. An example of this occurs in patients who have had irradiated salivary glands that gives rise to reduced function and limited salivary flow resulting in excessive and uncontrollable caries on the side of the irradiated gland (Pinna et al., 2015). Without the buffering capacity of saliva a selective group of low pH acidophilic commensal microbes (microbial diversification has been reduced) have free reign. In addition to buffering, salivary antimicrobials that include but are not limited to lactoferrin, lactoperoxidase, lysozyme, IgA, as well as salivary flow itself, are severely reduced and therefore this landscape (the enamel surface) is much more vulnerable to the activity of low pH adaptive members of the commensal microbiota.

In a second simple demonstration of the importance of saliva, subjects who abstained from brushing for a short period in order to accumulate tooth associated plaque, were asked to rinse with a 10% sucrose solution (Abelson and Mandel, 1981). An antimony electrode was placed on tooth-surface-associated-plaque-biofilm in order to measure the pH of the biofilm in real time. A dramatic rapid decline in the pH was seen immediately after the sucrose rinse. Thus, the plaque/biofilm pH dropped from a neutral pH of 7.0 to <6.0; however, shortly thereafter salivary buffering forced the pH to rise again, protecting the enamel surface of the tooth from demineralization due to acid production. To demonstrate the prominence of host related saliva on this plaque pH effect, the salivary ducts of the subjects were blocked prior to the sucrose rinse, which prevented saliva from contacting the tooth associated biofilm. This interference with salivary flow allowed for the immediate pH drop but prevented salivary buffering and as such the subsequent pH rise failed to occur. As a consequence, the pH of the plaque/biofilm dipped below the 5.5 critical pH such that demineralization could take place. This simple experiment is another clear example of the prominence of the host in Damage/Response Framework determination of oral disease.

### Summary of Causes of Dysbiosis and Dental Diseases

In summary, we have shown how classical common dental disease occur in the event of dysbiosis of the commensal microbiota initiated by an environmental stimulus that can cause a shift in the landscape ecology to the detriment of the host. In these examples there is no need for the addition of an exogenous pathogenic microbial species to cause disease. Rather classical dental “diseases” are more than likely to occur as a result of a shift in the activity and/or proportion of members of the commensal microbiota as a consequence of environmental changes. Thus, as in the case of most current infections, microbial interactions that have a damaging effect on the host are associated with the way in which the host manages the damage that can occur.

## Microbial and Immune Interactions in Health, Inflammation, and Autoimmune Diseases

### Brief Historical Prospective: General Immunology

Interest in immunology began in the late 1700's when small pox was raging as an epidemic in Europe. In 1796, Edward Jenner inoculated James Phipps with a scraping he obtained from a small pox lesion he removed from the arm of a dairy maiden who was infected by working with cows who had cowpox. This idea was derived from Benjamin Jesty who took scrapings from a cow (vacca) with a similar virus and inoculated his wife (Riedel, 2005). In 1875, Robert Koch inoculated the ear of a rabbit with blood of an animal that had anthrax. Shortly thereafter, Koch learned how to grow bacteria, validated the germ theory of disease, and began to establish the most advanced microbiology laboratory in the world (Williams et al., 2008).

Completely independently, Pasteur in 1879 began studying chicken cholera. In a serendipitous accident, Pasteur left nutrient broth intended to grow cholera toxin unattended in his laboratory over the summer. He then used the unattended broth, but left in his flask as an attenuated inoculum for chickens and found that they failed to get sick with cholera. In honor of Jenner, he called the process vaccination, but it was not until 1893 that Ehrlich identified the biological attributes as an anti-toxin material. In a contentious collaboration with von Behring and Shibasaburō, Ehrlich recognized that the anti-toxin for diphtheria was due to a soluble serum factor (Kaufmann, 2017).

In 1882, Eli Metchnikoff opened the door for research into white blood cells, phagocytosis, and innate immunity sparking the interest in cellular immunology (Gordon, 2008, 2016). Skipping ahead to 1939, Elvin Kabat, then at Columbia University, discovered that antibodies were gamma globulins (Kabat, 1983). In the 1960s, antibody structure was elucidated by Porter and Edelman, while Miller and Mitchell discovered B and T cell collaboration in functional antibody production in 1968 (Miller and Mitchell, 1968; Mitchell and Miller, 1968a,b; Raju, 1999; Sprent, 2017). A significant discovery of the activation of the innate immunity was made in the early 2000s by Beutler, Hoffman, and Steinman Beutler (2013).

### Brief Historical Prospective: Immunology Related to Oral Disease

The work describing immunology in oral diseases paralleled the work done in medicine. The pathogenesis of dental infections received a great deal of attention when the emphasis shifted from pyorrhea as a local disease to a disease directly related to causes of systemic diseases of unknown etiology (Hunter, 1900). A link between oral infections and arthritis, colitis, heart disease, and cancer of unknown etiology was made and received a great deal of attention (Colyer, 1902). This theory was known as the “focal theory of infection” and was supported by several prominent dental and medical researchers and academicians, one of which was R. L. Cecil, author of the well-known Cecil and Loeb “Textbook of Medicine,” first published in 1927 (Hunter, 1900, 1911; Billings, 1912; Cecil, 1929; Cecil and Angevine, 1938). During that period Rosenow, a prominent microbiologist, performed scientific experiments using animal models in efforts to show how microbes from the oral cavity provoked systemic infections (Rosenow, 1919, 1930). After several instances related to “extreme treatment” of human “dental infections,” whereby treatment resulted in extraction of all teeth, it was determined that the extreme treatment failed to result in any changes in overall systemic health. This approach put an end to the belief in this theory and the practice of “extreme treatment” was fortunately abandoned.

As for vaccinations related to pyorrhea, Beckwith et al. (1929) made efforts to inoculate animals with organisms isolated from pyorrheatic pockets (Beckwith et al., 1925, 1929). He compared reactions to heat attenuated plaque derived from humans to boiled plaque samples then inoculated into humans and rabbits. Several of the animals died in the heat-attenuated samples as opposed to the boiled samples suggesting some semi-viable toxic material (Beckwith et al., 1929). A series of studies were also initiated by Rosebury to develop caries vaccines although the investigators focused on lactobacillus as opposed to streptococci (Rosebury et al., 1929, 1934).

Major contributions to the study of oral immunology were made by the Alabama dental research group consisting of Drs. J. McGhee, Mestecky, and Michalek, also involving Dr. Per Brandtzaeg and Frederick Kraus. As prominent contributors to our understanding of the common immune mucosal system (CIMS), the group clearly illustrated the unification of IgA pathways when antigens were provided via vaccines to mucosal surfaces as compared to intramuscular inoculations (Mestecky et al., 1972, 1978, 2008; McGhee et al., 1987; Moldoveanu et al., 1995). Studies from this group revolved around development of a caries vaccine against *S. mutans*, which provided a unique understanding of mucosal immunity and ultimately showed differences in IgG, IgM, and IgA responses. Kiyono, also part of this group, provided a new method for separating dendritic cells and macrophages as antigen presenting cells in Peyer's patches and showed that oral delivery of antigens produced Ig isotype subset of Th2 type helper cells that induced IgA responsiveness (Kiyono and Fukuyama, 2004; Kiyono and Azegami, 2015).

### Recognition of Pathogens by the Innate Immune System

The vital observation that the induction of a strong immune response against purified proteins was dependent on the presence of microbial constituents, such as killed bacteria or bacterial extracts, famously called “the immunologist's dirty little secret” by Janeway (1989), gave birth to the term **adjuvant** (which in Latin means *adjuvare*, for “to help”). In the absence of infection, it is clear that adjuvants are partially required to activate innate receptors on sensor cells to aid T cells (lymphocytes). Sensor cells that detect infection and drive the production of inflammatory mediators include macrophages, neutrophils, and dendritic cells. Such cells express a number of innate recognition receptors that enable them to detect pathogens or the damage caused by them. These receptors are known as pattern recognition receptors (PRRs) and recognize simple molecular structures termed pathogen-associated molecular patterns (PAMPs), also called microbe-associated molecular patterns (MAMPs), which are components of many microorganisms, but not of the body's own cells (Yu et al., 2017; Negi et al., 2019). PAMPs come in various flavors and are expressed by different classes of bacteria, which engage several pattern recognition receptors (PRRs) (Table 2).

**Table 2:** Pattern recognition receptors (PRRs) and the associated pathogen-associated molecular patterns (PAMPs).

	**PRR**	**PAMP**	**References^**1**^**
Gram positive	TLR2, NOD2	Peptidoglycan (PGN)	a, d
	TLR2	Teichoic acid (TA)	b, c, d, e, i,
	TLR2/6	Lipoteichoic acid (LTA)	e, f, g
	TLR5, NAIP5, NAIP6, NLRC4	Flagella	d, zc
Gram negative	TLR4	Lipopolysaccharide (LPS)	d, h
	TLR2, NOD1, NOD2	PGN	d, j
	TLR5, NAIP5, NAIP6, NLRC4	Flagella	d, h
	TLR2	Porins	k, l
	NAIP2, NLRC4	Rod protein of Type III secretion system (T3SS)	m, n
**Genus-specific**			
Mycobacteria	TLR2, MARCO, MINCLE	Trehalose dimycolate (TDM)	o, p
	TLR2/4	Mycolic acid (MA)	q, r
	TLR2	Lipoarabinomannan (LAM)	s, t, u
	TLR2	Arabinogalactan (AG)	v
	TLR2, NOD2	Peptidoglycan (PGN)	w
	TLR4	Phosphatidylinositol mannose (PIM)	x
Mycoplasma	TLR2/6	Macrophage activating lipopeptide M161 antigen	d, y
Other	TLR9	CpG-DNA	z, za, zb

1 *a, Rosenzweig et al. (2011); b, Ribeiro et al. (2010); c, Kumar et al. (2013); d, Kumar et al. (2011); e, Takeuchi et al. (2000); f, Krutzik et al. (2003); g, Takeuchi et al. (2000); h, Takeuchi et al. (2002); i, Echchannaoui et al. (2002); j, Clarke et al. (2010); k, Singleton et al. (2005); l, Mukherjee et al. (2014); m, Kofoed and Vance (2011); n, Karki et al. (2018); o, Bowdish et al. (2009); p, Martinez et al. (2016); q, van Crevel et al. (2002); r, Harding and Boom (2010); s, Strohmeier and Fenton (1999); t, Hook et al. (2020); u, Gilleron et al. (2003); v, He et al. (2019); w, Girardin et al. (2003); x, Abel et al. (2002); y, Nishiguchi et al. (2001); z, Peter et al. (2009); za, Adamus and Kortylewski (2018); zb, Hausmann et al. (2020); zc, Lai et al. (2013)*.

While the first line of innate immune defense involves detection of PAMPs or MAMPs, danger-associated molecular patterns (DAMPs) are endogenous factors released upon cellular damage or tissue disruption (Kay et al., 2019). DAMPs released from oral and salivary tissue play an important role in progression of inflammatory and autoimmune disease. The signaling pathways of PAMPs and DAMPs intersect in the manifestation of diseases of the oral cavity, particularly in periodontal disease, oropharyngeal candidiasis, and Sjögren's Syndrome (De Lorenzo et al., 2018; Kay et al., 2019).

### Immunologic Signals Induced by Pathogen Recognition

Chemokines are chemotactic cytokines whose function is critical for the positioning of immune cells in tissues. They control release of innate immune cells from the bone marrow, as part of normal homeostasis, and as a result of infection and inflammation. They play a critical role in guiding innate immune effectors out of the circulation and into sites of injury or inflammation. In doing so, chemokines promote, and coordinate interactions between the innate and adaptive immune systems, thus ensuring optimal adaptive immune responses (Hao et al., 2010; Sokol and Luster, 2015). Neutrophils are the first cells to arrive at sites of infection, and they provide a front line of defense against bacterial infection. While most bacteria are readily killed by neutrophils, some bacterial pathogens have the capacity to circumvent destruction by these host leukocytes (Teng et al., 2017; Kobayashi et al., 2018). There is an elaborate cellular and cytokine presence at the gingival tissue interface and supporting oral mucosa, where an increased amount of neutrophils are recruited to the gingival crevice during inflammation, such as conditions found in gingivitis or periodontitis (Dutzan et al., 2016; Moutsopoulos and Konkel, 2018). Under normal conditions these neutrophils play an important role in microbial surveillance as well as in coordinating the overall immune response, in order to maintain oral health.

Evidence suggests that bacteria in biofilms, including those found in the supra- and subgingival plaque biofilm, are more resistant to the phagocytic activities of neutrophils and macrophages than non-biofilm bacteria (Ebersole et al., 2017; Liu et al., 2017). As a result, the sentinel cells that mediate the first line of the adaptive immune response, comprising dendritic cells, macrophages and mast cells, are called in for battle, scanning for the foreign invaders. The initial response is to destroy the invaders, followed by distress signals that are sent via cytokines and chemokines that recruit reinforcement of other effector cells to eliminate the remaining threat.

The adaptive immune system of the gastrointestinal tract has unique features that distinguish it from those of other organ systems. The most important adaptive immunity in the gut is humoral and is geared toward keeping the microbes of the lumen under control. This property is mediated by dimeric IgA antibodies, which are secreted into the lumen of the gut or found in the colostrum of mother's milk ingested by infants (Macpherson et al., 2018; Bryant and Thistle, 2020). IgA in the gut is critical in preventing commensals and pathogens from invading via the epithelial barrier of the mucosa. The preponderance of IgA in mucosal secretions is due to the fact that activated B cells in the gut undergo class switching to IgA producing B cells, which home to the gut. Cell mediated immunity against gut microbes are mediated by helper T cells, of which Th17 cells are the most abundant, even though Th1 and Th2 cells are also found. Regulatory T cells (Tregs) are most committed to maintaining tolerance to food antigens (Tordesillas and Berin, 2018), and to commensal microbial antigens (Nutsch and Hsieh, 2012).

Resident macrophages and dendritic cells are normally present in the gingiva and are important in defending the tissue barrier against bacterial insult. In response to microbial dysbiosis, the numbers of these cells increase (Delima and Van Dyke, 2003). In health, lymphocytes in the gingiva comprise few B cells and more prominent T cells. During disease, various B-cell and T-cell subsets increase significantly, where Th17 cells may promote pathogenesis. While little is known about specialization of Treg cells in the gingiva, it is clear that Tregs play critical roles in maintaining periodontal homeostasis (Glowacki et al., 2013; Moutsopoulos and Konkel, 2018). Unlike homeostatic oral Th17 cell accumulation, in a commensal-independent and IL-6-dependent manner, periodontitis-associated expansion of Th17 cells was dependent upon the local dysbiotic microbiome and required both IL-6 and IL-23 (Silva et al., 2015; Dutzan et al., 2018). Th17 cells secrete the IL-17 cytokines, which have pro-inflammatory activities in common with IL-1β, TNFα, and IL-22, and are important for immunity against extracellular bacteria (Miossec, 2009). Th17 cells are involved in the pathogenesis of several autoimmune and inflammatory disorders; in fact three IL-17 inhibitors have been approved for the treatment of psoriasis, psoriatic arthritis, and ankylosing spondylitis (Beringer and Miossec, 2019). As it relates to the oral cavity, IL-17A has been shown to stimulate the development of osteoclasts (osteoclastogenesis) in the presence of osteoblasts (Zhang et al., 2011), and expression of IL-17 has been observed in gingiva from patients with periodontitis (Cardoso et al., 2009). In an *A. actinomycetemcomitans*-induced rat model for periodontal disease prior to onset of bone resorption, upregulation of IL-17 in CD4+ T cells (2.8-fold) and B cells (2-fold) in lymph nodes from *A. actinomycetemcomitans*-infected rats was observed, as compared to control rats (Li et al., 2010; Tsiagbe and Fine, 2012).

The Th17/IL-17 response has been investigated as a therapeutic target. Resolvin E1 (RvE1), a product of the ω-3 polyunsaturated fatty acid eicosapentaenoic acid is known to be a potent pro-resolving lipid mediator that prevents chronic inflammation, osteoclastogenesis, and bone resorption by inhibiting IL-17-induced RANKL expression in osteoblasts and RANKL-induced osteoclast differentiation (Funaki et al., 2018). Its activities have made RvE1 a new therapeutic target of rheumatoid arthritis. Additionally, resolvins hold promise for treatment of periodontal disease and other inflammatory diseases, including type 2 diabetes and cardiovascular disease (Van Dyke, 2017).

Mucosal tissues, which are colonized by a dense and diverse microbiota of commensal bacteria, are often the initial sites of interaction with pathogenic microorganism (D'Aiuto et al., 2004; Ebersole et al., 2017). Macrophages efficiently recognize unique classes of microorganism-associated molecular patterns (MAMPs), which facilitate the avid uptake of the microbes by pattern recognition receptors (PRRs) (Lauvau and Glaichenhaus, 2004; Ebersole et al., 2017). In the “classical activation” (M1), the macrophages display an inflammatory function that leads to cytotoxicity, tissue injury, and fibrosis (Locati et al., 2013). The differentiation into M1 macrophage phenotype relates to host-derived IFN-γ, as an autocrine or paracrine factor, and lipopolysaccharide (Labonte et al., 2017). The “alternative activation” (M2a,b) process is driven by IL-4 and IL-13, which can be autocrine or paracrine, and is immunomodulatory in its control of tissue repair and cellular regeneration (Mantovani et al., 2013). Macrophage activation plays a large role in periodontal diseases. The outcome of antigen recognition is dependent on which functional subpopulations of macrophages are engaged. The oral pathogens *P. gingivalis* and *A. actinomycetemcomitans* were observed to induce M1-type cells, whereas oral commensal bacteria primarily elicited macrophage functions consistent with an M2 phenotype (Huang et al., 2016). The presence of relatively more M1 macrophages, compared to M2 macrophages in gingival tissue may be responsible for the development and progression of inflammation-induced tissue destruction, and modulating macrophage function may be a potential strategy for periodontal disease management (Zhou et al., 2019).

Immature dendritic cells (i.e., Langerhans's cells), which are endowed with the ability to capture antigen, are normally located in the gingival epithelium, while mature dendritic cells, which specialize in antigen presentation, tend to infiltrate specifically the lamina propria of the gingiva, an area enriched for CD4+ T cells (Jotwani et al., 2001). While much work is still needed to elucidate the role of dendritic cell subsets in periodontal disease, it is established that immature dendritic cells were more prevalent in aggressive periodontitis than chronic periodontitis (da Motta et al., 2016).

### Molecular Mimicry and Its Pathologic Consequences

The process of aging is characterized by quantitative modifications of the immune system, described as “immunosenescence,” which leads to increased susceptibility to infections, neoplasias, and autoimmune manifestations, primarily due to persistent antigenic stimulation and/or stress responses across the life span (Weng, 2006; McElhaney et al., 2012; Ebersole et al., 2016; Mancuso et al., 2018). This diminution in the ability to withstand antigenic stimuli or stressors is often accompanied by enhanced proinflammatory state, known as “inflammaging” (Ebersole et al., 2016; Fulop et al., 2018). This enhanced proinflammatory state is shared by the elderly who age with minimal morbidities (i.e., no comorbidities) and those who do not (Mari et al., 1995; Ebersole et al., 2016). This observation led to the hypothesis that a threshold exists beyond which an individual is driven toward unsuccessful aging (Shanley et al., 2009). The mechanisms that underlie inflammaging are not well-elucidated. One explanation put forward is that it is driven by changes in the numbers and frequencies of innate immune cells, or alteration in the expression of or signaling via PRRs (Baggio et al., 1998). A generalized age-associated decreased in toll-like receptor (TLR)-induced cytokine production has been observed (Canaday et al., 2010). With respect to periodontal disease, age-related decline in IL-6 induction in macrophages by *P. gingivalis* has been observed (Liang et al., 2009). In some individuals, age-related decline in TLR-dependent expression of costimulatory molecules CD80 and CD86 has been observed in monocytes, myeloid dendritic cells, and plasmacytoid dendritic cells (Qian et al., 2011; Sridharan et al., 2011; Ebersole et al., 2016).

Knowledge about age-related changes in the composition and phenotype of cells in the periodontium, which lead to alveolar bone resorption, gave birth to the concept of “osteoimmunology” (Feng and McDonald, 2011; Schett, 2016; Terashima and Takayanagi, 2018; Okamoto and Takayanagi, 2019). Age-related increases in RANK expression on osteoblast progenitors and RANKL expression in supporting mesenchymal stromal cells has been noted to result in a pro-osteoclastic environment, which potentially promotes bone resorption (Chung et al., 2014; Ebersole et al., 2016). While age-related enhancements in proinflammatory cytokines, such as prostaglandin E2, TNF-α, IL-1β, IL-6, and IL-17 are suggested to play significant roles in in enhancing osteoclastogenesis (Boyle et al., 2003; Ebersole et al., 2016), other molecules such as IFN-β, IL-4, IL-10 and chemokine axis of CCR4 and CCL22 dampen bone loss by a molecular feedback mechanism (Araujo-Pires et al., 2015; Ebersole et al., 2016).

Molecular mimicry of host proteins is an established strategy adopted by bacterial pathogens to interfere with and exploit host processes. Mimics within pathogens arise via two evolutionary mechanisms: (1) pathogen genomes can obtain host genes directly through lateral transfer or (2) through convergent or parallel evolution of a pathogenic protein toward resemblance of a host protein (Koonin et al., 2001; Stebbins and Galán, 2001; Elde and Malik, 2009; Doxey and McConkey, 2013). The Gram-negative bacterium *Helicobacter pylori* is a common bacterial pathogen that is responsible for widespread gastrointestinal morbidity worldwide and employs of a number of mechanisms of molecular mimicry (Kamboj et al., 2017). *H. pylori* colonizes the gastric mucosa in humans, and increases the risk of serious diseases such as gastric and duodenal ulcers, stomach cancers, and mucosa-associated lymphoid tissue lymphoma. *H. pylori* employs antigenic mimicry and possible deleterious effects due to the induction of immune response to the components common to these bacteria and the host (Chmiela and Gonciarz, 2017). *H. pylori*-related growth retardation in children is a noted phenomenon, however, it is poorly understood. Gastrointestinal microbiota, including *H. pylori* may produce antigens that mimic appetite-regulating peptides, resulting in the production of auto-antibodies, which modify the actions of key appetite-regulating peptides, such as alpha-melanocyte-stimulating hormone (α-MSH) (Fetissov et al., 2008). Polymorphisms of the host interleukins, including IL-1β, TNF-α, and cyclooxygenase-2 (COX2) have been suggested to increase the risk of infection and its severe consequences (Machado et al., 2003).

There is increasing support to the idea that gut dysbiosis, with an imbalanced state of microbiota, might be associated with the pathogenesis of autoimmune diseases, including rheumatoid arthritis (RA), systemic lupus erythematosus (SLE), ankylosing spondylitis (AS), and inflammatory bowel disease (IBD) (Kim et al., 2016). Tetracycline derivatives, such as doxycycline and minocycline, are safe and moderately effective disease modifying anti-rheumatic drugs in the treatment of early RA patients (Smith et al., 2011; Kim et al., 2016). Probiotics, which are live microorganisms that confer health benefits to the host and which have the potential to maintain a healthy microbial balance in the gut, have been tested. *Clostridium* consortium, *F. prausnitzii*, and *Bifidobacterium* have been tested to ameliorate IBD in the colitis model by inducing Treg cells and anti-inflammatory effects (Atarashi et al., 2011; Kim et al., 2016).

## Current and Future Methodologies for the Evaluation of Healthy and Dysbiotic Communities

The imbalance in the composition of oral microbiome can be directly linked to disease conditions. However, because of the overlap in the microbial communities, the difference between the healthy and disease states cannot be solely explained by the differences in the microbial composition. Thus, additional elements such as the functional activities of the microbiomes are needed to fully characterize and define the dysbiotic process. In this regard, recent omics studies have analyzed gene expression changes to analyze the functional activities and will be discussed below in detail.

### Omics Technologies

The omics approach encompasses various technologies applied to fields of research including genomics (and epigenomics), transcriptomics, proteomics, and metabolomics. The ultimate goal of these approaches is to design diagnostics to predict an individual's risk to develop disease and/or to determine whether or not specific treatments are suitable for the individual patient. A genomics approach has been widely used, especially in cancer diagnoses. Recently, genome-wide association studies (GWAS), and next generation exome and genome sequencing data have amassed a large set of DNA sequence variants that can be associated with diseases in humans (Olivier et al., 2019).

### Genome-Wide Association Studies and Periodontitis

Although GWAS have had modest success, (Offenbacher et al., 2016) supplemented the clinical data with biological intermediates of microbial burden and the local inflammatory response [gingival crevicular fluid (GCF) IL-1β] to derive periodontal complex traits (PCTs) for chronic periodontitis. Six PCTs were derived. PCT1 (loci CLEC19A, TRA, GGTA2P, TM9SF2, IFI16, and RBMS3) was characterized by a uniformly high pathogen load; PCT3 genetic variants of diacylglycerol kinase and inositol polyphosphate phosphatase, which are critically involved with regulating neutrophil function; and PCT5 (loci *SLC15A4*, PKP2, and SNRPN) were dominated by *Aggregatibacter actinomycetemcomitans* and *Porphyromonas gingivalis*. Several studies aiming to explore the distinct gene expression profiles and pathways unique to periodontitis have indeed confirmed the anticipatory differential expression involved in inflammation or bone resorption (Zhang et al., 2020). For example, pathways involved in cytokine and chemokine activities, B-cell receptor signaling, and defense and immunity proteins in both innate and adaptive immune responses, were reported to be among those most upregulated in periodontic gingival tissues when assessed using RNA sequencing.

It is unclear at present, despite much thought in to the subject of “Infectogenomics,” as to how host genetics helps to shape the healthy or dysbiotic microbiome in periodontitis (Kellam and Weiss, 2006). Recently, groups have been exploring how host genetic variants affect the colonization of bacteria, which affects the genetics-associated dysbiosis (Nibali et al., 2014, 2016). In a model proposed to explain this dysbiosis, it has been suggested that single-nucleotide polymorphism (SNP) variants may compromise genes that are associated with host pathways of bacterial sensing and recognition (Zhang et al., 2020). The advent of 16S ribosomal RNA gene sequencing has allowed the evaluation of phylogenetic relatedness of bacterial species. If the bacterial sensing and recognition pathways are affected and result in a dysbiotic biofilm, could this lead to a shift in the microbial taxa wherein the health-associated species are suppressed and periodontitis-associated bacteria dominate subgingival communities (Diaz et al., 2016)? Using 16S pyrosequencing of the microbiome of periodontitis patients and healthy controls, Griffen et al. (2012) reported the enrichment of 123 species abundant in periodontitis compared to only 53 species in the healthy state. This microbial shift in the proportion of existing species might result in a succession process of emergence of disease bacteria without replacing the health-associated species. As described below, it is possible that the shift in microbiota may also result in a metabolic shift in periodontitis patients.

### Metatranscriptomics

This omics method is a technology designed for the functional characterization of microbiomes using microarray or RNA sequencing technologies that reveals the taxonomic composition and active functions of a complex microbial community. It is unlike metagenomics, which shows the microbial DNA composition, and with an added bonus over previous methods since there is no PCR or primer/probe bias (Socransky et al., 1994; Kumar and Gupta, 2003; Colombo et al., 2009). With the well-characterized human microbiome, metatranscriptomics has facilitated many studies about the dysbiosis and disease. Discussed below are some such studies, which have helped us to understand the functional characterization of the microbiome.

#### Metatranscriptome and Caries

In an earlier study, focusing on the metatranscriptome during biofilm formation and before and after meal ingestion, the authors set out to identify the transcriptionally active portion of the supragingival microbial community in relation to the metagenome (Benitez-Paez et al., 2014). Clearly, from one individual the three most abundant genera from the metatranscriptome analysis of 24-hr plaque corresponded to *Actinomyces, Corynebacterium*, and *Neisseria*, whereas the most abundant genera from the metagenome were *Veillonella, Streptococcus*, and *Leptotrichia*. In the same study, the authors also analyzed the gene expression during plaque development and compared the genera during early (6–12 h) and mature biofilm (24–48 h) formation times. Interestingly, there was significantly (*p* < 0.0118) fewer genera in the early compared to the mature biofilm. Both *Streptococcus* and *Actinomyces*, known partners of coaggregation were found predominantly in the early time point (Kolenbrander et al., 1983). The bacterial activity during biofilm formation in healthy subjects was found to be person-specific. While in the early plaque samples, up regulation of genes involved in the carbohydrate metabolism, energy, vitamins, and amino acids were observed, in the mature biofilm the functional categories included ABC transporters, chemotaxis, and pilus assembly. One aspect of the study suggested that at least in some individuals there was no difference in the microbiota before or after a meal thus maintaining homeostasis and promoting dental health (Benitez-Paez et al., 2014).

#### Metatranscriptome and Periodontitis

The microbiota associated with periodontitis, a disease resulting in part from polymicrobial synergy and dysbiosis, has been classified into color coded complexes depending upon their accumulation in the periodontal pocket (Socransky et al., 1998; Lamont and Hajishengallis, 2015). Although the mean species (beta) diversity changes drastically between the disease and healthy states, higher alpha (average species) diversity and biomass appear to be associated with the disease community when the subgingival plaque was analyzed from chronic periodontitis patients and healthy subjects (Abusleme et al., 2013). In spite of the emergence of dominant taxa, the original health-associated community did not get replaced. Subsequent statistical analysis of two existing data sets, however, revealed a reduced alpha diversity associated with disease (Duran-Pinedo et al., 2014b; Yost et al., 2015; Ai et al., 2017). In a recent metatranscriptomic analysis, notably, the functional comparisons between healthy and generalized aggressive periodontitis sites revealed that upregulation of lysine fermentation, histidine degradation, and pyruvate metabolism is common among diseased individuals (Jorth et al., 2014). However, three metatranscriptomic surveys into the metabolic activity of chronic periodontal disease progression provided further insight that the conservation of the community functionality rather than the specific microbial effecters of disease exists (Duran-Pinedo et al., 2014a,b; Yost et al., 2015). A very recent work, examining the existing metatranscriptome datasets (Duran-Pinedo et al., 2014b; Jorth et al., 2014; Yost et al., 2015) to identify the commonly differentially expressed transcripts from both chronic and aggressive periodontitis patients and potential underlying RNA regulatory mechanisms behind the metabolic shifts, has revealed that many ncRNAs (both known and putative) may facilitate the metabolic shifts associated with periodontitis (Ram-Mohan and Meyer, 2020). Some of the notable highlights of this new analysis are: (1) Only a fraction of the differentially expressed transcripts originate from red/orange complex; (2) The differentially expressed transcripts from these Red/Orange complexes show the greatest magnitude of change; (3) Enrichment of genes generally grouped into the biological process of localization could be important in the establishment of red complex pathogens in the periodontal pocket to drive pathogenesis; (4) Most metabolic pathways enriched in disease state have multiple contributing species fulfilling the metabolic niche; and (5) By applying *de novo* pipelines on the differentially expressed genes, the authors identified several putative sense and anti-sense regulators of bacterial ribosomal proteins that could be associated with periodontitis. Additionally, seven novel antisense ncRNAs targeting ribosomal proteins may be involved in maintaining ribosomal protein stoichiometry during the disease associated metabolic shift (Ram-Mohan and Meyer, 2020). In summary, metatranscriptomics analyses suggest a common shift in metabolic signatures in disease vs. healthy communities with up-regulated processes including pyruvate fermentation, histidine degradation, amino acid metabolism, and TonB-dependent receptors.

### Proteomics

Simply put, proteomics is the methodology wherein proteins are identified in a sample. The key technology in proteome analysis is mass spectral analysis, although classical approaches have also utilized gel electrophoresis, liquid chromatography, and microarray. Mass spectrometry (MS)-based proteomics is a large-scale, high-throughput, systematic study, allowing for the comprehensive characterization of total protein in a sample, even with a limited sample volume or mass. Recent advances in the mass spectrometry platform utilize selective, multiple, and reaction monitoring to quantify precisely and reproducibly even low abundance proteins, which makes the technology even suitable for clinical use (Uzozie and Aebersold, 2018). Many of the current analyses also include protein-network analysis for enhancing the analytical outcome.

#### Proteomics and Caries

For caries research, the proteome of early pellicle (3 min) samples isolated from 12 caries-free and 12 caries-active patients was analyzed (Trautmann et al., 2019). Among the 1,188 proteins identified, there were 68 that were ubiquitous regardless of disease state. Quantitative analysis suggested that 23 proteins are potential caries-specific biomarkers. A higher extent of protein identifications might facilitate the future large-scale analyses to identify discrimination factors for the development of caries susceptibility tests (Trautmann et al., 2019).

In another study involving children with and without caries, using multiple reaction monitoring, unstimulated saliva from three groups of 10 children each with no, low, and high caries were analyzed (Wang et al., 2018). Among the 244 differentially expressed proteins, the authors selected 53 proteins, including mucins, histatin 1, cystatins, and basic salivary proline-rich protein 2, for further verification using multiple reaction monitoring assays. An interesting conclusion from this study is that there might be synergistic action among the proteins for caries resistance and for carcinogenicity. Unlike other proteome studies, this type of analysis might be used in the development of biomimetic, therapeutic peptides with preventive benefits for childhood caries (Wang et al., 2018).

#### Proteomics and Periodontitis

Recently, proteomics approaches have been applied to the diagnosis of periodontitis through the identification of protein biomarkers by comparing complete protein profiles in health vs. disease conditions using tandem mass spectrometry (Antezack et al., 2020). This study was designed to evaluate the protein profiles exclusive of the potential risk factors such as age, gender, hypertension, smoking habits, or diabetes by using a principal component analysis of top-ranking peaks and epidemiological data from a medical questionnaire from 141 subjects (67 periodontal and 74 control subjects). Samples from saliva, GCF, and plaque were used in the analysis, which showed that the composition in these samples reflected important differences in correlation with disease population (Papapanou et al., 2018). Although GCF showed a strong ability to distinguish periodontal patients with a sensitivity of 79.6 (0.188) and a specificity equal to 75.7 (0.195), saliva can be used as a simple diagnostic fluid for screening potential periodontal patients [sensitivity = 70.3% (0.211) and specificity = 77.8% (0.165)]. While other studies based on bacterial composition 16S rRNA analysis have shown *Prevotella* to be overabundant in healthy subjects, salivary microbiota such as *Porphyromonas, Tannerella, Desulfobulbus, Eubacterium, Phocaeicola*, and *Mogibacterium* were associated with periodontitis patients (Chen et al., 2018). One of the major outcomes of this mass spectral study is that periodontal diagnosis does not depend on a unique biomarker and that patients who are symptom-free can be screened at the early stage of disease without the need of clinical measurements such as pocket depth, plaque index and radiography (Papapanou et al., 2018). Thus, the mass spectroscopy analysis could be used as a simple, non-invasive, and rapid screening method on a large population within a short period of time to results (24–48 h).

In another study, Hartenbach et al., using a hybrid mass spectrometer LTQ Orbitrap Velos for proteomic analysis to obtain greater resolution, mass accuracy and sensitivity, were able to improve the qualitative and quantitative analyses of the salivary proteome (Hartenbach et al., 2020). The authors showed that a large range of salivary proteins with protective functions and associated with oral homeostasis were down-regulated in chronic periodontitis patients compared to individuals presenting periodontal health. In contrast, very few specific proteins such as salivary acidic proline-rich phosphoprotein, a submaxillary gland androgen-regulated protein, histatin-1, fatty acid binding protein, thioredoxin, and cystatin-SA were increased in chronic periodontitis or related to periodontal tissue destruction and inflammation. It is possible that a decrease of several proteins related to innate immune response and tissue integrity may determine the disease profile. These differences in salivary proteome profiles between periodontal health and periodontitis may contribute to the identification of disease indicators, and to the improvement of periodontal diagnosis and treatment.

What is the clinical relevance of these studies? While it could be argued that routine MS analysis might be useful, this technique also requires specialists for the analysis and might be time consuming. However, identifying a small subset of reliable changed proteins from these proteomic studies could lead to consistent identification of salivary proteins that could then be used in a chair-side test. In this regard, some recent studies have shown that S100A8 and S100A9 in GCF and saliva could be candidate biomarkers for periodontitis (Preiano et al., 2016; Shin et al., 2019). Fine et al., has shown that MIP1-α levels were 50-fold higher in aggressive periodontal patients' in saliva and can be used as a predictive marker for site-specific disease progression (Fine et al., 2009, 2014). MIP1-α has also been shown to be predictive in chronic periodontitis patient's GCF or saliva, as well as being one of a set of cytokines that influence the bacterial composition at the periodontal pocket, indicating a potential relationship to dysbiosis (Al-Sabbagh et al., 2012; Syndergaard et al., 2014; Zhou et al., 2017). Interestingly, this potential biomarker is elevated in many inflammatory and disease conditions that exhibit bone resorption, such as periodontitis, multiple myeloma, Sjögren syndrome, and rheumatoid arthritis (Bhavsar et al., 2015). Thus, a rapid, point-of-care test-kit using salivary MIP1-α, S100A8, and/or S100A9 could be a practical tool for diagnosis and reducing the risk of periodontitis and promotion of periodontal health (Buzalaf et al., 2020).

These studies provide a small sample of the potential of proteomics in diagnosing periodontal patients early on and to continue to follow the progress of the disease with minimal intervention and could become part of the routine dental visits. It should be noted, however, that the samples collected during a routine visit should be tested immediately after collection since storage of samples at −20°C for 2–3 months has been shown to alter the protein profiles (Preiano et al., 2016).

### Metabolomics

The high throughput analysis and biological understanding of how metabolites contribute to disease processes through metabolomics is an emerging field. We can appreciate the value of sequencing technologies in study of health and disease, however if one was interested in developing a bed or chair side point of care diagnostic device or test, metabolites would be a desirable analyte for detection.

#### Are There Bacterial Contributions to the Salivary Metabolome?

The salivary metabolomic studies are rapidly advancing and in this regard, it is prudent to understand the host-bacterial contributions when saliva is used as the diagnostic fluid. NMR has been utilized specifically for this purpose. For example, saliva is a diagnostic fluid that has been used in the metabolic profiling of various oral diseases such as caries. In this regard Gardner et al., have shown that there is significant contribution from the oral microbiota in unstimulated whole mouth saliva (Gardner et al., 2019). Specifically, when saliva samples of healthy volunteers were analyzed, using ^1^H-NMR, CFU enumeration, and principal component analysis, it was shown that whole saliva metabolites were positively correlated with bacterial load suggesting that the metabolite composition of whole saliva is more reflective of the oral microbiota than the underlying host metabolism, which was determined from plasma levels and parotid saliva. The whole saliva contained abundant short-chain fatty acids (acetate, propionate, butyrate, and formate) compared to parotic saliva or plasma. The authors conclude that whole saliva might be particularly useful in conjunction with NMR analysis to diagnose conditions reflective of dysbiosis. A comparative study of subjects with dental diseases might be useful for future dysbiosis studies (Gardner et al., 2019).

#### Metabolomics and Periodontitis

Like mass spectrometry, ^1^H-NMR has increasingly becoming a tool to study the metabolites associated with disease conditions. In a study that analyzed the NMR profiles of healthy control (*n* = 52) and post-treatment chronic periodontitis patients (*n* = 62) using un-stimulated saliva, 100 metabolites were characterized (Singh et al., 2019). Distinctive differences in the spectral data were subjected to multivariate analysis, which showed that there is an elevation in the concentration of statistically discriminant metabolites between control and diseased patient profiles. Among the 100 metabolites studied, 20 new metabolites indicate a bacterial population shift along with change in homeostasis, which might disturb the biofilm composition. Decreasing levels of N-acetylglucosamine along with pyruvate, glutamate, and ethane sulfonate support a shift from homeostatic to anaerobic conditions, a defining characteristic of the severity of chronic periodontitis (Aimetti et al., 2012). The Singh and Aimetti studies suggest that decreased levels of pyruvate and N-acetylglucosamine may be a signature in chronic periodontitis (Aimetti et al., 2012; Singh et al., 2019).

## Conclusion

Here in this review, we have presented how dysbiosis that occurs within host domains, and in particular the oral cavity, can affect disease. We have also outlined the current “omic” technologies that will allow researchers to examine the system as a whole in the future. Our particular emphasis has been on the role that commensals and pathobionts play in their interaction with the immune status of the host. It is apparent when considering the progress made in characterizing the oral microbiome and the oral immune environment that we are poised to begin to synthesize accurate models of the relative contributions of these components to disease.

While we have presented historical and new/advanced technologies that have been and will continue to be used to diagnose and assess the two most common dental diseases, caries and periodontal diseases we feel compelled to provide several cautionary notes worthy of comment. First, no technology can advance our understanding of disease until we have a unified, accepted, and clearly defined definition of health and disease. No matter how sophisticated the technology, poor and inconsistent definitions of disease will continue to lead to confusion rather than clarity. This is especially true since both microbial and host dysbiosis is so critical to shifts from health to disease in the oral cavity. Second, whatever technology we choose to utilize, we should make every effort to include data that is coordinated, comprehensive, and includes microbiology, host responsiveness, and disease progression or resolution. Since it is becoming clearer that oral disease has a relationship to overall health and well-being we need to extend our concerns related to how local oral diseases can effect overall general well-being. Third, since the diseases we study go through spurts of activity that vary from time to time, longitudinal rather than cross-sectional studies are preferred.

In this age of advanced technology there are several issues that need to be addressed if we are to advance the field of infectious diseases. In our field key issues relate to sampling, sample storage, analyte extraction and processing, data analysis, presentation and interpretation of results. As reports are published, the methods section has to be explicit in the description of the processes if we are to compare data and make meaningful conclusions among these large data sets. As an example to consider the level of detail needed the following must be addressed: when samples are taken is there a separation of supragingival and subgingival plaque, is the plaque collected by curette or paper point or some other device, are samples pooled or kept in independent vials, is there mention of the time of plaque collection, is it before, during or after disease has occurred? For analyte extraction what are the methods, has bead beating, sonication, biochemical methods been performed and for what time period, has the cell wall has been breached? Has the volume of analyte been standardized? With what technology platform are the samples analyzed? What pre and post-data analysis programs are used? Is data generated compared to a standardized database and if so which? What data analysis and statistical analysis is being conducted? Is Principal Component Analysis used as an initial determinant? How much is data tied to alpha and beta diversity, Shannon Diversity? Network Analysis of what kind? Linear Discriminant Analysis? These are a cross-section of questions that need to be described in each publication to reassure standardization of methodologies such that comparison of data is possible.

Finally, while we recognize that these criteria are demanding and difficult to accomplish our hope is that this review and the technologies presented herewith will inspire new ways of tackling persistent uncertainties. These obstinate questions have left us with huge gaps in our knowledge base in our efforts to both diagnose and treat oral diseases. While oral biologists do not stand alone in this dilemma perhaps this overview will be of some assistance in efforts to advance our understanding in the future.

## Author Contributions

CC, NR, VT, and DF contributed equally to conceptualizing, writing, and editing the manuscript. All authors contributed to the article and approved the submitted version.

## Conflict of Interest

The authors declare that the research was conducted in the absence of any commercial or financial relationships that could be construed as a potential conflict of interest.

## References

[B1] Abel B. Thieblemont N. Quesniaux V. J. Brown N. Mpagi J. Miyake K.. (2002). Toll-like receptor 4 expression is required to control chronic *Mycobacterium tuberculosis* infection in mice. J. Immunol. 169, 3155–3162. 10.4049/jimmunol.169.6.3155 12218133

[B2] Abelson D. C. Mandel I. D. (1981). The effect of saliva on plaque pH *in vivo*. J. Dent. Res. 60, 1634–1638. 10.1177/00220345810600090101 6943155

[B3] Abt M. C. Pamer E. G. (2014). Commensal bacteria mediated defenses against pathogens. Curr. Opin. Immunol. 29, 16–22. 10.1016/j.coi.2014.03.003 24727150 PMC4132187

[B4] Abusleme L. Dupuy A. K. Dutzan N. Silva N. Burleson J. A. Strausbaugh L. D.. (2013). The subgingival microbiome in health and periodontitis and its relationship with community biomass and inflammation. ISME J. 7, 1016–1025. 10.1038/ismej.2012.174 23303375 PMC3635234

[B5] Adamus T. Kortylewski M. (2018). The revival of CpG oligonucleotide-based cancer immunotherapies. Contemp. Oncol. 22, 56–60. 10.5114/wo.2018.73887 29628795 PMC5885070

[B6] Ai D. Huang R. Wen J. Li C. Zhu J. Xia L. C. (2017). Integrated metagenomic data analysis demonstrates that a loss of diversity in oral microbiota is associated with periodontitis. BMC Genomics 18:1041. 10.1186/s12864-016-3254-5 28198672 PMC5310281

[B7] Aimetti M. Cacciatore S. Graziano A. Tenori L. (2012). Metabonomic analysis of saliva reveals generalized chronic periodontitis signature. Metabolomics 8, 465–474. 10.1007/s11306-011-0331-2

[B8] Alm J. S. Swartz J. Björkstén B. Engstrand L. Engström J. Kühn I.. (2002). An anthroposophic lifestyle and intestinal microflora in infancy. Pediatr. Allergy Immunol. 13, 402–411. 10.1034/j.1399-3038.2002.01062.x 12485315

[B9] Al-Sabbagh M. Alladah A. Lin Y. Kryscio R. J. Thomas M. V. Ebersole J. L.. (2012). Bone remodeling-associated salivary biomarker MIP-1alpha distinguishes periodontal disease from health. J. Periodont. Res. 47, 389–395. 10.1111/j.1600-0765.2011.01445.x PMC3292624 22126530

[B10] Antezack A. Chaudet H. Tissot-Dupont H. Brouqui P. Monnet-Corti V. (2020). Rapid diagnosis of periodontitis, a feasibility study using MALDI-TOF mass spectrometry. PLoS ONE 15:e0230334. 10.1371/journal.pone.0230334 32168352 PMC7069628

[B11] Araujo-Pires A. C. Vieira A. E. Francisconi C. F. Biguetti C. C. Glowacki A. Yoshizawa S.. (2015). IL-4/CCL22/CCR4 axis controls regulatory T-cell migration that suppresses inflammatory bone loss in murine experimental periodontitis. J. Bone Miner. Res. 30, 412–422. 10.1002/jbmr.2376 25264308 PMC4542048

[B12] Atarashi K. Tanoue T. Shima T. Imaoka A. Kuwahara T. Momose Y.. (2011). Induction of colonic regulatory T cells by indigenous *Clostridium* species. Science 331, 337–341. 10.1126/science.1198469 21205640 PMC3969237

[B13] Baggio G. Donazzan S. Monti D. Mari D. Martini S. Gabelli C.. (1998). Lipoprotein(a) and lipoprotein profile in healthy centenarians: a reappraisal of vascular risk factors. FASEB J. 12, 433–437. 10.1096/fasebj.12.6.433 9535215

[B14] Beckwith T. D. Simonton F. V. Williams A. (1925). A histologic study of the gum in Pyorrhea^*^, ^**^Aided by grants from the Carnegie Corporation., the American Dental Association and the Associated Radiograph Laboratories.^*^From the Department of Bacteriology, University of California, Berkeley, University of California Dental School, San Francisco, and California Stomatological Research Group. J. Am. Dental Assoc. 12, 129–153. 10.14219/jada.archive.1925.0045

[B15] Beckwith T. D. Williams A. Rose E. T. (1929). The role of bacteria in pyorrhea. Med. J. Rec. 129, 333–336.

[B16] Benitez-Paez A. Belda-Ferre P. Simon-Soro A. Mira A. (2014). Microbiota diversity and gene expression dynamics in human oral biofilms. BMC Genomics 15:311. 10.1186/1471-2164-15-311 24767457 PMC4234424

[B17] Beringer A. Miossec P. (2019). Systemic effects of IL-17 in inflammatory arthritis. Nat. Rev. Rheumatol. 15, 491–501. 10.1038/s41584-019-0243-5 31227819

[B18] Berkowitz R. J. Jones P. (1985). Mouth-to-mouth transmission of the bacterium *Streptococcus mutans* between mother and child. Arch. Oral Biol. 30, 377–379. 10.1016/0003-9969(85)90014-7 3857909

[B19] Beutler B. (2013). Bruce Beutler: the persistent prospector. Interview by Ruth Williams. Circ. Res. 112, 751–754. 10.1161/CIRCRESAHA.113.301093 23449544

[B20] Bhavsar I. Miller C. Al-Sabbagh M. (2015). “Macrophage inflammatory protein-1 alpha (MIP-1 alpha)/CCL3: as a biomarker,” in General Methods in Biomarker Research and their Applications, Vol. 1, eds V. Preedy and V. Patel (Dordrecht: Springer), 223–249. 10.1007/978-94-007-7696-8_27

[B21] Billings F. (1912). Chronic focal infections and their etiologic relations to arthritis and nephritis. Archiv. Int. Med. 9, 484–498. 10.1001/archinte.1912.00060160087007

[B22] Bowdish D. M. Sakamoto K. Kim M. J. Kroos M. Mukhopadhyay S. Leifer C. A.. (2009). MARCO, TLR2, and CD14 are required for macrophage cytokine responses to mycobacterial trehalose dimycolate and *Mycobacterium tuberculosis*. PLoS Pathog. 5:e1000474. 10.1371/journal.ppat.1000474 19521507 PMC2688075

[B23] Boyle W. J. Simonet W. S. Lacey D. L. (2003). Osteoclast differentiation and activation. Nature 423, 337–342. 10.1038/nature01658 12748652

[B24] Brown K. A. Khanafer N. Daneman N. Fisman D. N. (2013). Meta-analysis of antibiotics and the risk of community-associated *Clostridium difficile* infection. Antimicrob. Agents Chemother. 57, 2326–2332. 10.1128/AAC.02176-12 23478961 PMC3632900

[B25] Bryant J. Thistle J. (2020). “Anatomy, colostrum,” in StatPearls (Treasure Island, FL: StatPearls Publishing.

[B26] Buffie C. G. Jarchum I. Equinda M. Lipuma L. Gobourne A. Viale A.. (2012). Profound alterations of intestinal microbiota following a single dose of clindamycin results in sustained susceptibility to *Clostridium difficile*-induced colitis. Infect. Immun. 80, 62–73. 10.1128/IAI.05496-11 22006564 PMC3255689

[B27] Buzalaf M. A. R. Ortiz A. C. Carvalho T. S. Fideles S. O. M. Araujo T. T.. (2020). Saliva as a diagnostic tool for dental caries, periodontal disease and cancer: is there a need for more biomarkers? Expert Rev. Mol. Diagn. 20, 543–555. 10.1080/14737159.2020.1743686 32223655

[B28] Canaday D. H. Amponsah N. A. Jones L. Tisch D. J. Hornick T. R. Ramachandra L. (2010). Influenza-induced production of interferon-alpha is defective in geriatric individuals. J. Clin. Immunol. 30, 373–383. 10.1007/s10875-010-9374-9 20182777 PMC2875067

[B29] Cardoso C. R. Garlet G. P. Crippa G. E. Rosa A. L. Júnior W. M. Rossi M. A.. (2009). Evidence of the presence of T helper type 17 cells in chronic lesions of human periodontal disease. Oral Microbiol. Immunol. 24, 1–6. 10.1111/j.1399-302X.2008.00463.x 19121062

[B30] Casadevall A. Pirofski L. A. (1999). Host-pathogen interactions: redefining the basic concepts of virulence and pathogenicity. Infect. Immun. 67, 3703–3713. 10.1128/IAI.67.8.3703-3713.1999 10417127 PMC96643

[B31] Casadevall A. Pirofski L. A. (2003). The damage-response framework of microbial pathogenesis. Nat. Rev. Microbiol. 1, 17–24. 10.1038/nrmicro732 15040176 PMC7097162

[B32] Casadevall A. Pirofski L. A. (2015). What is a host? Incorporating the microbiota into the damage-response framework. Infect. Immun. 83, 2–7. 10.1128/IAI.02627-14 25385796 PMC4288903

[B33] Casadevall A. Pirofski L. A. (2018). What Is a Host? Attributes of individual susceptibility. Infect. Immun. 86:e00636-17. 10.1128/IAI.00636-17 29084893 PMC5778373

[B34] Cecil R. L. (1929). A Text-book of Medicine: by American Authors. Philadelphia: Saunders.

[B35] Cecil R. L. Angevine D. M. (1938). Clinical and experimental observations of focal infection, with an analysis of 200 cases of rheumatoid arthritis. Ann. Intern. Med. 12, 577–584. 10.7326/0003-4819-12-5-577

[B36] Chen C. Hemme C. Beleno J. Shi Z. J. Ning D. Qin Y.. (2018). Oral microbiota of periodontal health and disease and their changes after nonsurgical periodontal therapy. ISME J. 12, 1210–1224. 10.1038/s41396-017-0037-1 29339824 PMC5932080

[B37] Chmiela M. Gonciarz W. (2017). Molecular mimicry in *Helicobacter pylori* infections. World J. Gastroenterol. 23, 3964–3977. 10.3748/wjg.v23.i22.3964 28652651 PMC5473117

[B38] Cho I. Blaser M. J. (2012). The human microbiome: at the interface of health and disease. Nat. Rev. Genet. 13, 260–270. 10.1038/nrg3182 22411464 PMC3418802

[B39] Chung P. L. Zhou S. Eslami B. Shen L. Leboff M. S. Glowacki J. (2014). Effect of age on regulation of human osteoclast differentiation. J. Cell. Biochem. 115, 1412–1419. 10.1002/jcb.24792 24700654 PMC4096781

[B40] Clarke J. K. (1924). On the bacterial factor in the ætiology of dental caries. Br. J. Exp. Pathol. 5, 141–147.

[B41] Clarke T. B. Davis K. M. Lysenko E. S. Zhou A. Y. Yu Y. Weiser J. N. (2010). Recognition of peptidoglycan from the microbiota by Nod1 enhances systemic innate immunity. Nat. Med. 16, 228–231. 10.1038/nm.2087 20081863 PMC4497535

[B42] Colombo A. P. Boches S. K. Cotton S. L. Goodson J. M. Kent R. Haffajee A. D.. (2009). Comparisons of subgingival microbial profiles of refractory periodontitis, severe periodontitis, and periodontal health using the human oral microbe identification microarray. J. Periodontol. 80, 1421–1432. 10.1902/jop.2009.090185 19722792 PMC3627366

[B43] Colyer S. (1902). Oral sepsis: and some of its effects. Dent. Rec. 20, 200–206.

[B44] Costello E. K. Stagaman K. Dethlefsen L. Bohannan B. J. Relman D. A. (2012). The application of ecological theory toward an understanding of the human microbiome. Science 336, 1255–1262. 10.1126/science.1224203 22674335 PMC4208626

[B45] Cox L. M. Yamanishi S. Sohn J. Alekseyenko A. V. Leung J. M. Cho I.. (2014). Altering the intestinal microbiota during a critical developmental window has lasting metabolic consequences. Cell 158, 705–721. 10.1016/j.cell.2014.05.052 25126780 PMC4134513

[B46] Cugini C. Klepac-Ceraj V. Rackaityte E. Riggs J. E. Davey M. E. (2013). *Porphyromonas gingivalis*: keeping the pathos out of the biont. J. Oral Microbiol. 5:1. 10.3402/jom.v5i0.19804 23565326 PMC3617648

[B47] Cullen C. M. Aneja K. K. Beyhan S. Cho C. E. Woloszynek S. Convertino M.. (2020). Emerging priorities for microbiome research. Front. Microbiol. 11:136. 10.3389/fmicb.2020.00136 32140140 PMC7042322

[B48] Curtis M. A. Diaz P. I. Van Dyke T. E. (2020). The role of the microbiota in periodontal disease. Periodontol. 2000 83, 14–25. 10.1111/prd.12296 32385883

[B49] da Motta R. J. Tirapelli C. Juns Da Silva R. Villafuerte K. R. Almeida L. Y. Ribeiro-Silva A.. (2016). Immature, but not mature, dendritic cells are more often present in aggressive periodontitis than chronic periodontitis: an immunohistochemical study. J. Periodontol. 87, 1499–1507. 10.1902/jop.2016.150729 27389962

[B50] D'Aiuto F. Parkar M. Andreou G. Suvan J. Brett P. M. Ready D.. (2004). Periodontitis and systemic inflammation: control of the local infection is associated with a reduction in serum inflammatory markers. J. Dent. Res. 83, 156–160. 10.1177/154405910408300214 14742655

[B51] Dashper S. G. Reynolds E. C. (1996). Lactic acid excretion by *Streptococcus mutans*. Microbiology 142, 33–39. 10.1099/13500872-142-1-33 33657745

[B52] Dawes C. (2003). What is the critical pH and why does a tooth dissolve in acid? J. Can. Dent. Assoc. 69, 722–724. 14653937

[B53] De Lorenzo G. Ferrari S. Cervone F. Okun E. (2018). Extracellular DAMPs in plants and mammals: immunity, tissue damage and repair. Trends Immunol. 39, 937–950. 10.1016/j.it.2018.09.006 30293747

[B54] Delima A. J. Van Dyke T. E. (2003). Origin and function of the cellular components in gingival crevice fluid. Periodontol. 2000 31, 55–76. 10.1034/j.1600-0757.2003.03105.x 12656996

[B55] Dethlefsen L. Huse S. Sogin M. L. Relman D. A. (2008). The pervasive effects of an antibiotic on the human gut microbiota, as revealed by deep 16S rRNA sequencing. PLoS Biol. 6:e280. 10.1371/journal.pbio.0060280 19018661 PMC2586385

[B56] Dewhirst F. E. Chen T. Izard J. Paster B. J. Tanner A. C. Yu W. H.. (2010). The human oral microbiome. J. Bacteriol. 192, 5002–5017. 10.1128/JB.00542-10 20656903 PMC2944498

[B57] Diaz P. I. Hoare A. Hong B. Y. (2016). Subgingival microbiome shifts and community dynamics in periodontal diseases. J. Calif. Dent. Assoc. 44, 421–435. 27514154

[B58] Dominguez-Bello M. G. Godoy-Vitorino F. Knight R. Blaser M. J. (2019). Role of the microbiome in human development. Gut 68, 1108–1114. 10.1136/gutjnl-2018-317503 30670574 PMC6580755

[B59] Dorey R. B. Theodosiou A. A. Read R. C. Jones C. E. (2019). The nonpathogenic commensal *Neisseria*: friends and foes in infectious disease. Curr. Opin. Infect. Dis. 32, 490–496. 10.1097/QCO.0000000000000585 31356239

[B60] Doxey A. C. McConkey B. J. (2013). Prediction of molecular mimicry candidates in human pathogenic bacteria. Virulence 4, 453–466. 10.4161/viru.25180 23715053 PMC5359739

[B61] Duguid R. (1985). *In-vitro* acid production by the oral bacterium *Streptococcus mutans* 10449 in various concentrations of glucose, fructose and sucrose. Arch. Oral Biol. 30, 319–324. 10.1016/0003-9969(85)90004-4 3857902

[B62] Duran-Pinedo A. E. Baker V. D. Frias-Lopez J. (2014a). The periodontal pathogen *Porphyromonas gingivalis* induces expression of transposases and cell death of *Streptococcus mitis* in a biofilm model. Infect. Immun. 82, 3374–3382. 10.1128/IAI.01976-14 24866802 PMC4136200

[B63] Duran-Pinedo A. E. Chen T. Teles R. Starr J. R. Wang X. Krishnan K.. (2014b). Community-wide transcriptome of the oral microbiome in subjects with and without periodontitis. ISME J. 8, 1659–1672. 10.1038/ismej.2014.23 24599074 PMC4817619

[B64] Dutzan N. Kajikawa T. Abusleme L. Greenwell-Wild T. Zuazo C. E. Ikeuchi T.. (2018). A dysbiotic microbiome triggers TH17 cells to mediate oral mucosal immunopathology in mice and humans. Sci. Transl. Med. 10:eaat0797. 10.1126/scitranslmed.aat0797 30333238 PMC6330016

[B65] Dutzan N. Konkel J. E. Greenwell-Wild T. Moutsopoulos N. M. (2016). Characterization of the human immune cell network at the gingival barrier. Mucosal Immunol. 9, 1163–1172. 10.1038/mi.2015.136 26732676 PMC4820049

[B66] Ebersole J. L. Dawson D. 3rd Emecen-Huja P. Nagarajan R. Howard K. Grady M. E. Thompson K.. (2017). The periodontal war: microbes and immunity. Periodontol. 2000 75, 52–115. 10.1111/prd.12222 28758303

[B67] Ebersole J. L. Graves C. L. Gonzalez O. A. Dawson D. III. Morford L. A. Huja P. E.. (2016). Aging, inflammation, immunity and periodontal disease. Periodontol. 2000 72, 54–75. 10.1111/prd.12135 27501491

[B68] Echchannaoui H. Frei K. Schnell C. Leib S. L. Zimmerli W. Landmann R. (2002). Toll-like receptor 2-deficient mice are highly susceptible to *Streptococcus pneumoniae* meningitis because of reduced bacterial clearing and enhanced inflammation. J. Infect. Dis. 186, 798–806. 10.1086/342845 12198614

[B69] Elde N. C. Malik H. S. (2009). The evolutionary conundrum of pathogen mimicry. Nat. Rev. Microbiol. 7, 787–797. 10.1038/nrmicro2222 19806153

[B70] Ellekilde M. Selfjord E. Larsen C. S. Jakesevic M. Rune I. Tranberg B.. (2014). Transfer of gut microbiota from lean and obese mice to antibiotic-treated mice. Sci. Rep. 4:5922. 10.1038/srep05922 25082483 PMC4118149

[B71] Englander H. R. Keyes P. H. (1964). Failure to induce dental caries in hamsters by transfer of plaque and carious debris from humans. J. Dent. Res. 43:626. 10.1177/00220345640430041701 14183351

[B72] Esberg A. Barone A. Eriksson L. Lif Holgerson P. Teneberg S. Johansson I. (2020). *Corynebacterium matruchotii* demography and adhesion determinants in the oral cavity of healthy individuals. Microorganisms 8:1780. 10.3390/microorganisms8111780 33202844 PMC7697164

[B73] Falk P. G. Hooper L. V. Midtvedt T. Gordon J. I. (1998). Creating and maintaining the gastrointestinal ecosystem: what we know and need to know from gnotobiology. Microbiol. Mol. Biol. Rev. 62, 1157–1170. 10.1128/MMBR.62.4.1157-1170.1998 9841668 PMC98942

[B74] Feng X. McDonald J. M. (2011). Disorders of bone remodeling. Annu. Rev. Pathol. 6, 121–145. 10.1146/annurev-pathol-011110-130203 20936937 PMC3571087

[B75] Fetissov S. O. Hamze Sinno M. Coëffier M. Bole-Feysot C. Ducrotté P. Hökfelt T.. (2008). Autoantibodies against appetite-regulating peptide hormones and neuropeptides: putative modulation by gut microflora. Nutrition 24, 348–359. 10.1016/j.nut.2007.12.006 18262391 PMC7126273

[B76] Fine D. H. (1995). Chemical agents to prevent and regulate plaque development. Periodontol. 2000 8, 87–107. 10.1111/j.1600-0757.1995.tb00047.x 9567948

[B77] Fine D. H. (2006). Dr. Theodor Rosebury: grandfather of modern oral microbiology. J. Dent. Res. 85, 990–995. 10.1177/154405910608501103 17062737

[B78] Fine D. H. Korik I. Furgang D. Myers R. Olshan A. Barnett M. L.. (1996). Assessing pre-procedural subgingival irrigation and rinsing with an antiseptic mouthrinse to reduce bacteremia. JADA 127, 641–646. 10.14219/jada.archive.1996.0276 8642144

[B79] Fine D. H. Markowitz K. Fairlie K. Tischio-Bereski D. Ferrandiz J. Godboley D.. (2014). Macrophage inflammatory protein-1alpha shows predictive value as a risk marker for subjects and sites vulnerable to bone loss in a longitudinal model of aggressive periodontitis. PLoS ONE 9:e98541. 10.1371/journal.pone.0098541 24901458 PMC4047026

[B80] Fine D. H. Markowitz K. Fairlie K. Tischio-Bereski D. Ferrendiz J. Furgang D.. (2013). A consortium of *Aggregatibacter actinomycetemcomitans, Streptococcus parasanguinis*, and *Filifactor alocis* is present in sites prior to bone loss in a longitudinal study of localized aggressive periodontitis. J. Clin. Microbiol. 51, 2850–2861. 10.1128/JCM.00729-13 23784124 PMC3754677

[B81] Fine D. H. Markowitz K. Furgang D. Fairlie K. Ferrandiz J. Nasri C.. (2009). Macrophage inflammatory protein-1alpha: a salivary biomarker of bone loss in a longitudinal cohort study of children at risk for aggressive periodontal disease? J. Periodontol. 80, 106–113. 10.1902/jop.2009.080296 19228096

[B82] Fitzgerald R. J. Keyes P. H. (1960). Demonstration of the etiologic role of streptococci in experimental caries in the hamster. J. Am. Dent. Assoc. 61, 9–19. 10.14219/jada.archive.1960.0138 13823312

[B83] Fitzgerald R. J. Keyes P. H. (1963). Ecologic factors in dental caries. the fate of antibiotic-resistant cariogenic streptococci in hamsters. Am. J. Pathol. 42, 759–772. 13945368 PMC1949716

[B84] Foster K. R. Schluter J. Coyte K. Z. Rakoff-Nahoum S. (2017). The evolution of the host microbiome as an ecosystem on a leash. Nature 548, 43–51. 10.1038/nature23292 28770836 PMC5749636

[B85] Fulop T. Witkowski J. M. Olivieri F. Larbi A. (2018). The integration of inflammaging in age-related diseases. Semin. Immunol. 40, 17–35. 10.1016/j.smim.2018.09.003 30287177

[B86] Funaki Y. Hasegawa Y. Okazaki R. Yamasaki A. Sueda Y. Yamamoto A.. (2018). Resolvin E1 inhibits osteoclastogenesis and bone resorption by suppressing IL-17-induced RANKL expression in osteoblasts and RANKL-induced osteoclast differentiation. Yonago Acta Med. 61, 8–18. 10.33160/yam.2018.03.002 29599617 PMC5871721

[B87] Gardner A. Parkes H. G. So P.-W. Carpenter G. H. (2019). Determining bacterial and host contributions to the human salivary metabolome. J. Oral Microbiol. 11:1617014. 10.1080/20002297.2019.1617014 PMC7610937 34109015

[B88] Gilleron M. Quesniaux V. F. Puzo G. (2003). Acylation state of the phosphatidylinositol hexamannosides from *Mycobacterium bovis* bacillus Calmette Guerin and mycobacterium tuberculosis H37Rv and its implication in Toll-like receptor response. J. Biol. Chem. 278, 29880–29889. 10.1074/jbc.M303446200 12775723

[B89] Girardin S. E. Boneca I. G. Carneiro L. A. Antignac A. Jéhanno M. Viala J.. (2003). Nod1 detects a unique muropeptide from gram-negative bacterial peptidoglycan. Science 300, 1584–1587. 10.1126/science.1084677 12791997

[B90] Glowacki A. J. Yoshizawa S. Jhunjhunwala S. Vieira A. E. Garlet G. P. Sfeir C.. (2013). Prevention of inflammation-mediated bone loss in murine and canine periodontal disease via recruitment of regulatory lymphocytes. Proc. Natl. Acad. Sci. U.S.A. 110, 18525–18530. 10.1073/pnas.1302829110 24167272 PMC3831997

[B91] Gordon S. (2008). Elie Metchnikoff: father of natural immunity. Eur. J. Immunol. 38, 3257–3264. 10.1002/eji.200838855 19039772

[B92] Gordon S. (2016). Elie Metchnikoff, the Man and the Myth. J. Innate Immun. 8, 223–227. 10.1159/000443331 26836137 PMC6738810

[B93] Griffen A. L. Beall C. J. Campbell J. H. Firestone N. D. Kumar P. S. Yang Z. K.. (2012). Distinct and complex bacterial profiles in human periodontitis and health revealed by 16S pyrosequencing. ISME J. 6, 1176–1185. 10.1038/ismej.2011.191 22170420 PMC3358035

[B94] Haffajee A. D. Patel M. Socransky S. S. (2008). Microbiological changes associated with four different periodontal therapies for the treatment of chronic periodontitis. Oral Microbiol. Immunol. 23, 148–157. 10.1111/j.1399-302X.2007.00403.x 18279183

[B95] Hagenfeld D. Koch R. Junemann S. Prior K. Harks I. Eickholz P.. (2018). Do we treat our patients or rather periodontal microbes with adjunctive antibiotics in periodontal therapy? A 16S rDNA microbial community analysis. PLoS ONE 13:e0195534. 10.1371/journal.pone.0195534 29668720 PMC5906003

[B96] Hajishengallis G. Moutsopoulos N. M. (2014). Etiology of leukocyte adhesion deficiency-associated periodontitis revisited: not a raging infection but a raging inflammatory response. Expert Rev. Clin. Immunol. 10, 973–975. 10.1586/1744666X.2014.929944 24931458 PMC4117468

[B97] Hajishengallis G. Moutsopoulos N. M. (2016). Role of bacteria in leukocyte adhesion deficiency-associated periodontitis. Microb. Pathog. 94, 21–26. 10.1016/j.micpath.2015.09.003 26375893 PMC4791199

[B98] Hanna S. Etzioni A. (2012). Leukocyte adhesion deficiencies. Ann. N. Y. Acad. Sci. 1250, 50–55. 10.1111/j.1749-6632.2011.06389.x 22276660

[B99] Hao J. Liu R. Piao W. Zhou Q. Vollmer T. L. Campagnolo D. I.. (2010). Central nervous system (CNS)-resident natural killer cells suppress Th17 responses and CNS autoimmune pathology. J. Exp. Med. 207, 1907–1921. 10.1084/jem.20092749 20696699 PMC2931174

[B100] Harding C. V. Boom W. H. (2010). Regulation of antigen presentation by *Mycobacterium tuberculosis*: a role for Toll-like receptors. Nat. Rev. Microbiol. 8, 296–307. 10.1038/nrmicro2321 20234378 PMC3037727

[B101] Hartenbach F. Velasquez E. Nogueira F. C. S. Domont G. B. Ferreira E. Colombo A. P. V. (2020). Proteomic analysis of whole saliva in chronic periodontitis. J. Proteomics 213:103602. 10.1016/j.jprot.2019.103602 31809901

[B102] Hausmann A. Böck D. Geiser P. Berthold D. L. Fattinger S. A. Furter M.. (2020). Intestinal epithelial NAIP/NLRC4 restricts systemic dissemination of the adapted pathogen *Salmonella Typhimurium* due to site-specific bacterial PAMP expression. Mucosal Immunol. 13, 530–544. 10.1038/s41385-019-0247-0 31953493 PMC7181392

[B103] He J. Fu W. Zhao S. Zhang C. Sun T. Jiang T. (2019). Lack of MSMEG_6281, a peptidoglycan amidase, affects cell wall integrity and virulence of *Mycobacterium smegmatis*. Microb. Pathog. 128, 405–413. 10.1016/j.micpath.2019.01.013 30685363

[B104] Hojo K. Nagaoka S. Ohshima T. Maeda N. (2009). Bacterial interactions in dental biofilm development. J. Dent. Res. 88, 982–990. 10.1177/0022034509346811 19828884

[B105] Hook J. S. Cao M. Weng K. Kinnare N. Moreland J. G. (2020). Mycobacterium tuberculosis lipoarabinomannan activates human neutrophils via a TLR2/1 mechanism distinct from Pam(3)CSK(4). J. Immunol. 204, 671–681. 10.4049/jimmunol.1900919 31871022

[B106] Hooks K. B. O'Malley M. A. (2017). Dysbiosis and its discontents. MBio 8:e01492-17. 10.1128/mBio.01492-17 29018121 PMC5635691

[B107] Hsiao E. Y. Mcbride S. W. Hsien S. Sharon G. Hyde E. R. Mccue T.. (2013). Microbiota modulate behavioral and physiological abnormalities associated with neurodevelopmental disorders. Cell 155, 1451–1463. 10.1016/j.cell.2013.11.024 24315484 PMC3897394

[B108] Huang C. B. Alimova Y. Ebersole J. L. (2016). Macrophage polarization in response to oral commensals and pathogens. Pathog. Dis. 74:ftw011. 10.1093/femspd/ftw011 26884502 PMC5975235

[B109] Human Microbiome Project C. (2012). Structure, function and diversity of the healthy human microbiome. Nature 486, 207–214. 10.1038/nature11234 22699609 PMC3564958

[B110] Hunter W. (1900). Oral sepsis as a cause of disease. Br. Med. J. 2, 215–216. 10.1136/bmj.2.2065.215 20759127 PMC2462945

[B111] Hunter W. (1911). The Role of Sepsis and Antisepsis in Medicine and the Importance of Oral Sepsis as Its Chief Cause. The Dental Register LXV, 579. PMC6984846 33702091

[B112] Jakubovics N. S. Gill S. R. Iobst S. E. Vickerman M. M. Kolenbrander P. E. (2008a). Regulation of gene expression in a mixed-genus community: stabilized arginine biosynthesis in *Streptococcus gordonii* by coaggregation with *Actinomyces naeslundii*. J. Bacteriol. 190, 3646–3657. 10.1128/JB.00088-08 18359813 PMC2395002

[B113] Jakubovics N. S. Gill S. R. Vickerman M. M. Kolenbrander P. E. (2008b). Role of hydrogen peroxide in competition and cooperation between *Streptococcus gordonii* and *Actinomyces naeslundii*. FEMS Microbiol. Ecol. 66, 637–644. 10.1111/j.1574-6941.2008.00585.x 18785881 PMC2820160

[B114] James J. (1994). Van Leeuwenhoek's discoveries of 1677–1678: a look too far. Micron 25, 1–4. 10.1016/0968-4328(94)90050-7

[B115] Janeway C. A. Jr. (1989). Approaching the asymptote? Evolution and revolution in immunology. Cold Spring Harb. Symp. Quant. Biol. 54(Pt 1), 1–13. 10.1101/SQB.1989.054.01.003 2700931

[B116] Jorth P. Turner K. H. Gumus P. Nizam N. Buduneli N. Whiteley M. (2014). Metatranscriptomics of the human oral microbiome during health and disease. MBio 5:e01012-14. 10.1128/mBio.01012-14 24692635 PMC3977359

[B117] Jotwani R. Palucka A. K. Al-Quotub M. Nouri-Shirazi M. Kim J. Bell D.. (2001). Mature dendritic cells infiltrate the T cell-rich region of oral mucosa in chronic periodontitis: *in situ, in vivo*, and *in vitro* studies. J. Immunol. 167, 4693–4700. 10.4049/jimmunol.167.8.4693 11591800 PMC3739284

[B118] Kabat E. A. (1983). Getting started 50 years ago–experiences, perspectives, and problems of the first 21 years. Annu. Rev. Immunol. 1, 1–32. 10.1146/annurev.iy.01.040183.000245 6399974

[B119] Kamboj A. K. Cotter T. G. Oxentenko A. S. (2017). *Helicobacter pylori*: the past, present, and future in management. Mayo Clin. Proc. 92, 599–604. 10.1016/j.mayocp.2016.11.017 28209367

[B120] Karki R. Lee E. Place D. Samir P. Mavuluri J. Sharma B. R.. (2018). IRF8 regulates transcription of naips for NLRC4 inflammasome activation. Cell 173, 920.e13–933.e13. 10.1016/j.cell.2018.02.055 29576451 PMC5935577

[B121] Kaufmann S. H. E. (2017). Remembering Emil von Behring: from tetanus treatment to antibody cooperation with phagocytes. MBio 8:e00117-17. 10.1128/mBio.00117-17 28246359 PMC5347343

[B122] Kay J. G. Kramer J. M. Visser M. B. (2019). Danger signals in oral cavity-related diseases. J. Leukoc. Biol. 106, 193–200. 10.1002/JLB.4MIR1118-439R 30776147 PMC6597288

[B123] Kellam P. Weiss R. A. (2006). Infectogenomics: insights from the host genome into infectious diseases. Cell 124, 695–697. 10.1016/j.cell.2006.02.003 16497580 PMC7119327

[B124] Kennedy E. A. King K. Y. Baldridge M. T. (2018). Mouse microbiota models: comparing germ-free mice and antibiotics treatment as tools for modifying gut bacteria. Front. Physiol. 9:1534. 10.3389/fphys.2018.01534 30429801 PMC6220354

[B125] Keyes P. H. Fitzgerald R. J. (1962). Dental caries in the Syrian hamster. IX. Arch. Oral Biol. 7, 267–277. 10.1016/0003-9969(62)90017-1 14455466

[B126] Kianoush N. Adler C. J. Nguyen K. A. Browne G. V. Simonian M. Hunter N. (2014). Bacterial profile of dentine caries and the impact of pH on bacterial population diversity. PLoS ONE 9:e92940. 10.1371/journal.pone.0092940 24675997 PMC3968045

[B127] Kim D. Yoo S. A. Kim W. U. (2016). Gut microbiota in autoimmunity: potential for clinical applications. Arch. Pharm. Res. 39, 1565–1576. 10.1007/s12272-016-0796-7 27444041

[B128] Kim W. J. Higashi D. Goytia M. Rendon M. A. Pilligua-Lucas M. Bronnimann M.. (2019). Commensal *Neisseria* kill *Neisseria gonorrhoeae* through a DNA-dependent mechanism. Cell Host Microbe 26, 228.e8–239.e8. 10.1016/j.chom.2019.07.003 31378677 PMC6728082

[B129] Kiyono H. Azegami T. (2015). The mucosal immune system: from dentistry to vaccine development. Proc. Jpn. Acad. Ser. B Phys. Biol. Sci. 91, 423–439. 10.2183/pjab.91.423 26460320 PMC4729857

[B130] Kiyono H. Fukuyama S. (2004). NALT- versus Peyer's-patch-mediated mucosal immunity. Nat. Rev. Immunol. 4, 699–710. 10.1038/nri1439 15343369 PMC7097243

[B131] Kobayashi S. D. Malachowa N. Deleo F. R. (2018). Neutrophils and bacterial immune evasion. J. Innate Immun. 10, 432–441. 10.1159/000487756 29642066 PMC6784029

[B132] Koch R. (1893). Ueber den augenblicklichen Stand der bakteriologischen Choleradiagnose. Zeitschrift für Hygiene und Infektionskrankheiten 14, 319–338. 10.1007/BF02284324

[B133] Kofoed E. M. Vance R. E. (2011). Innate immune recognition of bacterial ligands by NAIPs determines inflammasome specificity. Nature 477, 592–595. 10.1038/nature10394 21874021 PMC3184209

[B134] Kolenbrander P. E. (2000). Oral microbial communities: biofilms, interactions, and genetic systems. Annu. Rev. Microbiol. 54, 413–437. 10.1146/annurev.micro.54.1.413 11018133

[B135] Kolenbrander P. E. Andersen R. N. Blehert D. S. Egland P. G. Foster J. S. Palmer R. J. Jr. (2002). Communication among oral bacteria. Microbiol. Mol. Biol. Rev. 66, 486–505. 10.1128/MMBR.66.3.486-505.2002 12209001 PMC120797

[B136] Kolenbrander P. E. Inouye Y. Holdeman L. V. (1983). New *Actinomyces* and *Streptococcus* coaggregation groups among human oral isolates from the same site. Infect. Immun. 41, 501–506. 10.1128/IAI.41.2.501-506.1983 6409807 PMC264669

[B137] Kolenbrander P. E. Palmer R. J. Jr. Rickard A. H. Jakubovics N. S. Chalmers N. I. Diaz P. I. (2006). Bacterial interactions and successions during plaque development. Periodontol. 2000 42, 47–79. 10.1111/j.1600-0757.2006.00187.x 16930306

[B138] Kong X. Liu J. Cetinbas M. Sadreyev R. Koh M. Huang H.. (2019). New and preliminary evidence on altered oral and gut microbiota in individuals with Autism Spectrum Disorder (ASD): implications for ASD diagnosis and subtyping based on microbial biomarkers. Nutrients 11:2128. 10.3390/nu11092128 31489949 PMC6770733

[B139] Koonin E. V. Makarova K. S. Aravind L. (2001). Horizontal gene transfer in prokaryotes: quantification and classification. Annu. Rev. Microbiol. 55, 709–742. 10.1146/annurev.micro.55.1.709 11544372 PMC4781227

[B140] Kreth J. Merritt J. Shi W. Qi F. (2005). Competition and coexistence between *Streptococcus mutans* and *Streptococcus sanguinis* in the dental biofilm. J. Bacteriol. 187, 7193–7203. 10.1128/JB.187.21.7193-7203.2005 16237003 PMC1272965

[B141] Kritchevsky B. Seguin P. (1918). The pathogenesis and treatment of pyorrhea alveolaris. Dental Cosmos Monthly Rec. Dental Sci. 60, 781–784.

[B142] Krutzik S. R. Ochoa M. T. Sieling P. A. Uematsu S. Ng Y. W. Legaspi A.. (2003). Activation and regulation of Toll-like receptors 2 and 1 in human leprosy. Nat. Med. 9, 525–532. 10.1038/nm864 12692544

[B143] Kumar H. Kawai T. Akira S. (2011). Pathogen recognition by the innate immune system. Int. Rev. Immunol. 30, 16–34. 10.3109/08830185.2010.529976 21235323

[B144] Kumar P. Gupta K. C. (2003). A rapid method for the construction of oligonucleotide arrays. Bioconjug. Chem. 14, 507–512. 10.1021/bc025646q 12757372

[B145] Kumar S. Ingle H. Prasad D. V. Kumar H. (2013). Recognition of bacterial infection by innate immune sensors. Crit. Rev. Microbiol. 39, 229–246. 10.3109/1040841X.2012.706249 22866947

[B146] Labonte A. C. Sung S. J. Jennelle L. T. Dandekar A. P. Hahn Y. S. (2017). Expression of scavenger receptor-AI promotes alternative activation of murine macrophages to limit hepatic inflammation and fibrosis. Hepatology 65, 32–43. 10.1002/hep.28873 27770558 PMC5191952

[B147] Lai M. A. Quarles E. K. López-Yglesias A. H. Zhao X. Hajjar A. M. Smith K. D. (2013). Innate immune detection of flagellin positively and negatively regulates salmonella infection. PLoS ONE 8:e72047. 10.1371/journal.pone.0072047 23977202 PMC3747147

[B148] Lamell C. W. Griffen A. L. Mcclellan D. L. Leys E. J. (2000). Acquisition and colonization stability of *Actinobacillus actinomycetemcomitans* and *Porphyromonas gingivalis* in children. J. Clin. Microbiol. 38, 1196–1169. 10.1128/JCM.38.3.1196-1199.2000 10699021 PMC86374

[B149] Lamont R. J. Hajishengallis G. (2015). Polymicrobial synergy and dysbiosis in inflammatory disease. Trends Mol. Med. 21, 172–183. 10.1016/j.molmed.2014.11.004 25498392 PMC4352384

[B150] Lamont R. J. Koo H. Hajishengallis G. (2018). The oral microbiota: dynamic communities and host interactions. Nat. Rev. Microbiol. 16, 745–759. 10.1038/s41579-018-0089-x 30301974 PMC6278837

[B151] Lane N. (2015). The unseen world: reflections on Leeuwenhoek (1677) ‘concerning little animals'. Philos. Trans. R. Soc. Lond. B Biol. Sci. 370, 1–10. 10.1098/rstb.2014.0344 25750239 PMC4360124

[B152] Lang N. P. Kiel R. A. Anderhalden K. (1983). Clinical and microbiological effects of subgingival restorations with overhanging or clinically perfect margins. J. Clin. Periodontol. 10, 563–578. 10.1111/j.1600-051X.1983.tb01295.x 6581173

[B153] Lauvau G. Glaichenhaus N. (2004). Mini-review: presentation of pathogen-derived antigens *in vivo*. Eur. J. Immunol. 34, 913–920. 10.1002/eji.200424944 15048701

[B154] Li J. Helmerhorst E. J. Leone C. W. Troxler R. F. Yaskell T. Haffajee A. D.. (2004). Identification of early microbial colonizers in human dental biofilm. J. Appl. Microbiol. 97, 1311–1318. 10.1111/j.1365-2672.2004.02420.x 15546422

[B155] Li Y. Messina C. Bendaoud M. Fine D. H. Schreiner H. Tsiagbe V. K. (2010). Adaptive immune response in osteoclastic bone resorption induced by orally administered *Aggregatibacter actinomycetemcomitans* in a rat model of periodontal disease. Mol. Oral Microbiol. 25, 275–292. 10.1111/j.2041-1014.2010.00576.x 20618701

[B156] Liang S. Domon H. Hosur K. B. Wang M. Hajishengallis G. (2009). Age-related alterations in innate immune receptor expression and ability of macrophages to respond to pathogen challenge *in vitro*. Mech. Ageing Dev. 130, 538–546. 10.1016/j.mad.2009.06.006 19559723 PMC2717634

[B157] Liu J. Sun L. Liu W. Guo L. Liu Z. Wei X.. (2017). A nuclease from *Streptococcus mutans* facilitates biofilm dispersal and escape from killing by neutrophil extracellular traps. Front. Cell. Infect. Microbiol. 7:97. 10.3389/fcimb.2017.00097 28401067 PMC5368189

[B158] Lloyd-Price J. Abu-Ali G. Huttenhower C. (2016). The healthy human microbiome. Genome Med. 8:51. 10.1186/s13073-016-0307-y 27122046 PMC4848870

[B159] Locati M. Mantovani A. Sica A. (2013). Macrophage activation and polarization as an adaptive component of innate immunity. Adv. Immunol. 120, 163–184. 10.1016/B978-0-12-417028-5.00006-5 24070384

[B160] Loe H. Theilade E. Jensen S. B. Schiott C. R. (1967). Experimental gingivitis in man. 3. Influence of antibiotics on gingival plaque development. J. Periodontal. Res. 2, 282–289. 10.1111/j.1600-0765.1967.tb01901.x 4249983

[B161] Loesche W. J. (1976). Chemotherapy of dental plaque infections. Oral Sci. Rev. 9, 65–107. 1067529

[B162] Loesche W. J. (1993). Bacterial mediators in periodontal disease. Clin Infect Dis. 16(Suppl. 4), S203–S210. 10.1093/clinids/16.Supplement_4.S203 8324120

[B163] Loo V. G. Bourgault A. M. Poirier L. Lamothe F. Michaud S. Turgeon N.. (2011). Host and pathogen factors for *Clostridium difficile* infection and colonization. N. Engl. J. Med. 365, 1693–1703. 10.1056/NEJMoa1012413 22047560

[B164] Lu J. Claud E. C. (2019). Connection between gut microbiome and brain development in preterm infants. Dev. Psychobiol. 61, 739–751. 10.1002/dev.21806 30460694 PMC6728148

[B165] Lu J. Lu L. Yu Y. Cluette-Brown J. Martin C. R. Claud E. C. (2018). Effects of intestinal microbiota on brain development in humanized gnotobiotic mice. Sci. Rep. 8:5443. 10.1038/s41598-018-23692-w 29615691 PMC5882882

[B166] Macdonald J. B. Sutton R. M. Knoll M. L. Madlener E. M. Grainger R. M. (1956). The pathogenic components of an experimental fusospirochetal infection. J. Infect. Dis. 98, 15–20. 10.1093/infdis/98.1.15 13295619

[B167] Machado J. C. Figueiredo C. Canedo P. Pharoah P. Carvalho R. Nabais S.. (2003). A proinflammatory genetic profile increases the risk for chronic atrophic gastritis and gastric carcinoma. Gastroenterology 125, 364–371. 10.1016/S0016-5085(03)00899-0 12891537

[B168] Macpherson A. J. Harris N. L. (2004). Interactions between commensal intestinal bacteria and the immune system. Nat. Rev. Immunol. 4, 478–485. 10.1038/nri1373 15173836

[B169] Macpherson A. J. Yilmaz B. Limenitakis J. P. Ganal-Vonarburg S. C. (2018). IgA function in relation to the intestinal microbiota. Annu. Rev. Immunol. 36, 359–381. 10.1146/annurev-immunol-042617-053238 29400985

[B170] Mancuso S. Carlisi M. Santoro M. Napolitano M. Raso S. Siragusa S. (2018). Immunosenescence and lymphomagenesis. Immun. Ageing 15:22. 10.1186/s12979-018-0130-y 30258468 PMC6151062

[B171] Mantovani A. Biswas S. K. Galdiero M. R. Sica A. Locati M. (2013). Macrophage plasticity and polarization in tissue repair and remodelling. J. Pathol. 229, 176–185. 10.1002/path.4133 23096265

[B172] Marcinkiewicz J. Strus M. Pasich E. (2013). Antibiotic resistance: a “dark side” of biofilmassociated chronic infections. Pol. Arch. Med. 123, 309–313. 10.20452/pamw.1780 23828150

[B173] Mari D. Mannucci P. M. Coppola R. Bottasso B. Bauer K. A. Rosenberg R. D. (1995). Hypercoagulability in centenarians: the paradox of successful aging. Blood 85, 3144–3149. 10.1182/blood.V85.11.3144.bloodjournal85113144 7756646

[B174] Marsh P. D. (1994). Microbial ecology of dental plaque and its significance in health and disease. Adv. Dent. Res. 8, 263–271. 10.1177/08959374940080022001 7865085

[B175] Marsh P. D. Zaura E. (2017). Dental biofilm: ecological interactions in health and disease. J Clin Periodontol. 44(Suppl. 18), S12–S22. 10.1111/jcpe.12679 28266111

[B176] Martinez N. Ketheesan N. West K. Vallerskog T. Kornfeld H. (2016). Impaired recognition of *Mycobacterium tuberculosis* by alveolar macrophages from diabetic mice. J. Infect. Dis. 214, 1629–1637. 10.1093/infdis/jiw436 27630197 PMC5144731

[B177] Mazmanian S. K. Round J. L. Kasper D. L. (2008). A microbial symbiosis factor prevents intestinal inflammatory disease. Nature 453, 620–625. 10.1038/nature07008 18509436

[B178] McElhaney J. E. Zhou X. Talbot H. K. Soethout E. Bleackley R. C. Granville D. J.. (2012). The unmet need in the elderly: how immunosenescence, CMV infection, co-morbidities and frailty are a challenge for the development of more effective influenza vaccines. Vaccine 30, 2060–2067. 10.1016/j.vaccine.2012.01.015 22289511 PMC3345132

[B179] McGhee J. R. Kiyono H. Michalek S. M. Mestecky J. (1987). Enteric immunization reveals a T cell network for IgA responses and suggests that humans possess a common mucosal immune system. Antonie Van Leeuwenhoek 53, 537–543. 10.1007/BF00415514 3329827

[B180] Mestecky J. Kulhavy R. Kraus F. W. (1972). Studies on human secretory immunoglobulin A. II. Subunit structure. J. Immunol. 108, 738–747. 4110988

[B181] Mestecky J. Mcghee J. R. Michalek S. M. Arnold R. R. Crago S. S. Babb J. L. (1978). Concept of the local and common mucosal immune response. Adv. Exp. Med. Biol. 107, 185–192. 10.1007/978-1-4684-3369-2_22 742482

[B182] Mestecky J. Nguyen H. Czerkinsky C. Kiyono H. (2008). Oral immunization: an update. Curr. Opin. Gastroenterol. 24, 713–719. 10.1097/MOG.0b013e32830d58be 19122521

[B183] Miller J. F. Mitchell G. F. (1968). Cell to cell interaction in the immune response. I. Hemolysin-forming cells in neonatally thymectomized mice reconstituted with thymus or thoracic duct lymphocytes. J. Exp. Med. 128, 801–820. 10.1084/jem.128.4.801 5691985 PMC2138540

[B184] Miller W. D. (1890). The Micro-Organisms of the Human Mouth: The Local and General Diseases which are Caused by Them. Birmingham, AL: Classics of Dentistry Library.

[B185] Miossec P. (2009). IL-17 and Th17 cells in human inflammatory diseases. Microbes Infect. 11, 625–630. 10.1016/j.micinf.2009.04.003 19371791

[B186] Mira A. Simon-Soro A. Curtis M. A. (2017). Role of microbial communities in the pathogenesis of periodontal diseases and caries. J. Clin. Periodontol. 44(Suppl. 18), S23–S38. 10.1111/jcpe.12671 28266108

[B187] Mitchell G. F. Miller J. F. (1968a). Cell to cell interaction in the immune response. II. The source of hemolysin-forming cells in irradiated mice given bone marrow and thymus or thoracic duct lymphocytes. J. Exp. Med. 128, 821–837. 10.1084/jem.128.4.821 5691986 PMC2138546

[B188] Mitchell G. F. Miller J. F. (1968b). Immunological activity of thymus and thoracic-duct lymphocytes. Proc. Natl. Acad. Sci. U.S.A. 59, 296–303. 10.1073/pnas.59.1.296 4873344 PMC286035

[B189] Moldoveanu Z. Russell M. W. Wu H. Y. Huang W. Q. Compans R. W. Mestecky J. (1995). Compartmentalization within the common mucosal immune system. Adv. Exp. Med. Biol. 371A, 97–101. 10.1007/978-1-4615-1941-6_17 8526027

[B190] Moore W. E. C. Moore L. V. H. (1994). The bacteria of periodontal disease. Periodontol. 2000 5, 66–77. 10.1111/j.1600-0757.1994.tb00019.x 9673163

[B191] Moutsopoulos N. M. Chalmers N. I. Barb J. J. Abusleme L. Greenwell-Wild T. Dutzan N.. (2015). Subgingival microbial communities in Leukocyte Adhesion Deficiency and their relationship with local immunopathology. PLoS Pathog. 11:e1004698. 10.1371/journal.ppat.1004698 25741691 PMC4351202

[B192] Moutsopoulos N. M. Konkel J. E. (2018). Tissue-specific immunity at the oral mucosal barrier. Trends Immunol. 39, 276–287. 10.1016/j.it.2017.08.005 28923364 PMC5843496

[B193] Mueller N. T. Bakacs E. Combellick J. Grigoryan Z. Dominguez-Bello M. G. (2015). The infant microbiome development: mom matters. Trends Mol. Med. 21, 109–117. 10.1016/j.molmed.2014.12.002 25578246 PMC4464665

[B194] Mukherjee S. Sinha D. Ghosh A. K. Biswas T. (2014). Bacterial ligand stimulates TLR2-dependent chemokines of colon cell. Immunobiology 219, 350–356. 10.1016/j.imbio.2013.12.002 24565410

[B195] Negi S. Das D. K. Pahari S. Nadeem S. Agrewala J. N. (2019). Potential role of gut microbiota in induction and regulation of innate immune memory. Front. Immunol. 10:2441. 10.3389/fimmu.2019.02441 31749793 PMC6842962

[B196] Nibali L. Di Iorio A. Onabolu O. Lin G. H. (2016). Periodontal infectogenomics: systematic review of associations between host genetic variants and subgingival microbial detection. J. Clin. Periodontol. 43, 889–900. 10.1111/jcpe.12600 27440507

[B197] Nibali L. Henderson B. Sadiq S. T. Donos N. (2014). Genetic dysbiosis: the role of microbial insults in chronic inflammatory diseases. J. Oral Microbiol. 6, 1–10. 10.3402/jom.v6.22962 24578801 PMC3936111

[B198] Nishiguchi M. Matsumoto M. Takao T. Hoshino M. Shimonishi Y. Tsuji S.. (2001). *Mycoplasma fermentans* lipoprotein M161Ag-induced cell activation is mediated by Toll-like receptor 2: role of N-terminal hydrophobic portion in its multiple functions. J. Immunol. 166, 2610–2616. 10.4049/jimmunol.166.4.2610 11160323

[B199] Nutsch K. M. Hsieh C. S. (2012). T cell tolerance and immunity to commensal bacteria. Curr. Opin. Immunol. 24, 385–391. 10.1016/j.coi.2012.04.009 22613090 PMC3423487

[B200] Offenbacher S. Divaris K. Barros S. P. Moss K. L. Marchesan J. T. Morelli T.. (2016). Genome-wide association study of biologically informed periodontal complex traits offers novel insights into the genetic basis of periodontal disease. Hum. Mol. Genet. 25, 2113–2129. 10.1093/hmg/ddw069 26962152 PMC5062586

[B201] Okamoto K. Takayanagi H. (2019). Osteoimmunology. Cold Spring Harb. Perspect. Med. 9, 1–28. 10.1101/cshperspect.a031245 PMC6314075 29610150

[B202] Olivier M. Asmis R. Hawkins G. A. Howard T. D. Cox L. A. (2019). The need for multi-omics biomarker signatures in precision medicine. Int. J. Mol. Sci. 20:4781. 10.3390/ijms20194781 31561483 PMC6801754

[B203] O'Malley M. A. (2007). The nineteenth century roots of ‘everything is everywhere'. Nat. Rev. Microbiol. 5, 647–651. 10.1038/nrmicro1711 17603517

[B204] Page R. C. Schroeder H. E. (1976). Pathogenesis of inflammatory periodontal disease. A summary of current work. Lab. Invest. 34, 235–249. 765622

[B205] Pandey A. Cleary D. W. Laver J. R. Gorringe A. Deasy A. M. Dale A. P.. (2018). Microevolution of *Neisseria lactamica* during nasopharyngeal colonisation induced by controlled human infection. Nat. Commun. 9:4753. 10.1038/s41467-018-07235-5 30420631 PMC6232127

[B206] Papapanou P. N. Sanz M. Buduneli N. Dietrich T. Feres M. Fine D. H.. (2018). Periodontitis: consensus report of workgroup 2 of the 2017 world workshop on the classification of periodontal and peri-implant diseases and conditions. J. Periodontol. 89(Suppl. 1), S173–S182. 10.1002/JPER.17-0721 29926951

[B207] Park O. J. Yi H. Jeon J. H. Kang S. S. Koo K. T. Kum K. Y.. (2015). Pyrosequencing analysis of subgingival microbiota in distinct periodontal conditions. J. Dent. Res. 94, 921–927. 10.1177/0022034515583531 25904141

[B208] Peter M. E. Kubarenko A. V. Weber A. N. Dalpke A. H. (2009). Identification of an N-terminal recognition site in TLR9 that contributes to CpG-DNA-mediated receptor activation. J. Immunol. 182, 7690–7697. 10.4049/jimmunol.0900819 19494293

[B209] Petersen C. Round J. L. (2014). Defining dysbiosis and its influence on host immunity and disease. Cell. Microbiol. 16, 1024–1033. 10.1111/cmi.12308 24798552 PMC4143175

[B210] Pinna R. Campus G. Cumbo E. Mura I. Milia E. (2015). Xerostomia induced by radiotherapy: an overview of the physiopathology, clinical evidence, and management of the oral damage. Ther. Clin. Risk Manag. 11, 171–188. 10.2147/TCRM.S70652 25691810 PMC4325830

[B211] Pirofski L. A. Casadevall A. (2008). The damage-response framework of microbial pathogenesis and infectious diseases. Adv. Exp. Med. Biol. 635, 135–146. 10.1007/978-0-387-09550-9_11 18841709 PMC7123708

[B212] Polechová J. Storch D. (2019). “Ecological niche,” in Encyclopedia of Ecology, 2nd Edn, ed B. Fath (Oxford: Elsevier), 72–80. 10.1016/B978-0-12-409548-9.11113-3

[B213] Preiano M. Maggisano G. Lombardo N. Montalcini T. Paduano S. Pelaia G.. (2016). Influence of storage conditions on MALDI-TOF MS profiling of gingival crevicular fluid: Implications on the role of S100A8 and S100A9 for clinical and proteomic based diagnostic investigations. Proteomics 16, 1033–1045. 10.1002/pmic.201500328 26711623

[B214] Qian F. Wang X. Zhang L. Lin A. Zhao H. Fikrig E.. (2011). Impaired interferon signaling in dendritic cells from older donors infected *in vitro* with West Nile virus. J. Infect. Dis. 203, 1415–1424. 10.1093/infdis/jir048 21398396 PMC3080893

[B215] Raju T. N. (1999). The Nobel chronicles. 1972: gerald M Edelman (b 1929) and Rodney R Porter (1917-85). Lancet 354:1040. 10.1016/S0140-6736(05)76658-7 10501404

[B216] Ram-Mohan N. Meyer M. M. (2020). Comparative metatranscriptomics of periodontitis supports a common polymicrobial shift in metabolic function and identifies novel putative disease-associated ncRNAs. Front. Microbiol. 11:482. 10.3389/fmicb.2020.00482 32328037 PMC7160235

[B217] Relman D. A. (2012). The human microbiome: ecosystem resilience and health. Nutr Rev. 70(Suppl. 1), S2–S9. 10.1111/j.1753-4887.2012.00489.x 22861804 PMC3422777

[B218] Ribeiro C. M. Hermsen T. Taverne-Thiele A. J. Savelkoul H. F. Wiegertjes G. F. (2010). Evolution of recognition of ligands from Gram-positive bacteria: similarities and differences in the TLR2-mediated response between mammalian vertebrates and teleost fish. J. Immunol. 184, 2355–2368. 10.4049/jimmunol.0900990 20118281

[B219] Riedel S. (2005). Edward Jenner and the history of smallpox and vaccination. Proc. Bayl. Univ. Med. Cent. 18, 21–25. 10.1080/08998280.2005.11928028 16200144 PMC1200696

[B220] Rook G. A. (2010). 99th Dahlem conference on infection, inflammation and chronic inflammatory disorders: darwinian medicine and the ‘hygiene' or ‘old friends' hypothesis. Clin. Exp. Immunol. 160, 70–79. 10.1111/j.1365-2249.2010.04133.x 20415854 PMC2841838

[B221] Rook G. A. Raison C. L. Lowry C. A. (2014). Microbial ‘old friends', immunoregulation and socioeconomic status. Clin. Exp. Immunol. 177, 1–12. 10.1111/cei.12269 24401109 PMC4089149

[B222] Rook G. A. W. Lowry C. A. Raison C.L. (2013). Microbial “Old Friends” immunoregulation and stress resilience. Evol. Med. Public Health 2013, 46–64. 10.1093/emph/eot004 24481186 PMC3868387

[B223] Rosebury T. Foley G. Greenberg S. (1934). Studies of lactobacilli in relation to caries in rats. 2. Attempt to immunize rats, on cariesproducing diets, against lactobacilli. J. Dent. Res. 14, 231–232.

[B224] Rosebury T. Linton R. W. Buchbinder L. (1929). A comparative study of dental aciduric organisms and *Lactobacillus Acidophilus*. J. Bacteriol. 18, 395–412. 10.1128/JB.18.6.395-412.1929 16559404 PMC375090

[B225] Rosenow E. C. (1919). Studies on elective localization focal infection with special reference to oral sepsis'. J. Dent. Res. 1, 205–267. 10.1177/00220345190010030101 PMC7845802 33703338

[B226] Rosenow E. C. (1930). Elective localization of streptococci. Br. Med. J. 1, 1100–1101. 10.1136/bmj.1.3623.1100 20775513 PMC2313521

[B227] Rosenzweig H. L. Galster K. Vance E. E. Ensign-Lewis J. Nunez G. Davey M. P.. (2011). NOD2 deficiency results in increased susceptibility to peptidoglycan-induced uveitis in mice. Invest. Ophthalmol. Vis. Sci. 52, 4106–4112. 10.1167/iovs.10-6263 21296813 PMC3175939

[B228] Round J. L. Mazmanian S. K. (2009). The gut microbiota shapes intestinal immune responses during health and disease. Nat. Rev. Immunol. 9, 313–323. 10.1038/nri2515 19343057 PMC4095778

[B229] Schett G. (2016). [Osteoimmunology]. Z. Rheumatol. 75, 531–533. 10.1007/s00393-016-0144-9 27444621

[B230] Shafquat A. Joice R. Simmons S. L. Huttenhower C. (2014). Functional and phylogenetic assembly of microbial communities in the human microbiome. Trends Microbiol. 22, 261–266. 10.1016/j.tim.2014.01.011 24618403 PMC4008634

[B231] Shanley D. P. Aw D. Manley N. R. Palmer D. B. (2009). An evolutionary perspective on the mechanisms of immunosenescence. Trends Immunol. 30, 374–381. 10.1016/j.it.2009.05.001 19541538

[B232] Sharma H. Turner C. E. Siggins M. K. El-Bahrawy M. Pichon B. Kearns A.. (2019). Toxic shock syndrome toxin 1 evaluation and antibiotic impact in a transgenic model of staphylococcal soft tissue infection. mSphere 4:e00665-19. 10.1128/mSphere.00665-19 31597722 PMC6796978

[B233] Shin M. S. Kim Y. G. Shin Y. J. Ko B. J. Kim S. Kim H. D. (2019). Deep sequencing salivary proteins for periodontitis using proteomics. Clin. Oral Investig. 23, 3571–3580. 10.1007/s00784-018-2779-1 30554327

[B234] Silva L. M. Brenchley L. Moutsopoulos N. M. (2019). Primary immunodeficiencies reveal the essential role of tissue neutrophils in periodontitis. Immunol. Rev. 287, 226–235. 10.1111/imr.12724 30565245 PMC7015146

[B235] Silva N. Abusleme L. Bravo D. Dutzan N. Garcia-Sesnich J. Vernal R.. (2015). Host response mechanisms in periodontal diseases. J. Appl. Oral Sci. 23, 329–355. 10.1590/1678-775720140259 26221929 PMC4510669

[B236] Singh M. P. Saxena M. Saimbi C. S. Siddiqui M. H. Roy R. (2019). Post-periodontal surgery propounds early repair salivary biomarkers by (1)H NMR based metabolomics. Metabolomics 15:141. 10.1007/s11306-019-1593-3 31612356

[B237] Singleton T. E. Massari P. Wetzler L. M. (2005). Neisserial porin-induced dendritic cell activation is MyD88 and TLR2 dependent. J. Immunol. 174, 3545–3550. 10.4049/jimmunol.174.6.3545 15749891

[B238] Smith C. J. Sayles H. Mikuls T. R. Michaud K. (2011). Minocycline and doxycycline therapy in community patients with rheumatoid arthritis: prescribing patterns, patient-level determinants of use, and patient-reported side effects. Arthritis Res. Ther. 13:R168. 10.1186/ar3491 22008667 PMC3308103

[B239] Socransky S. S. (1979). Criteria for the infectious agents in dental caries and periodontal disease. J. Clin. Periodontol. 6, 16–21. 10.1111/j.1600-051X.1979.tb02114.x 295292

[B240] Socransky S. S. Haffajee A. D. (1991). Microbial mechanisms in the pathogenesis of destructive periodontal diseases: a critical assessment. J. Periodont. Res. 26, 195–212. 10.1111/j.1600-0765.1991.tb01646.x 1831843

[B241] Socransky S. S. Haffajee A. D. (1994). Evidence of bacterial etiology: a historical perspective. Periodontol. 2000 5, 7–25. 10.1111/j.1600-0757.1994.tb00016.x 9673160

[B242] Socransky S. S. Haffajee A. D. Cugini M. A. Smith C. Kent R. L. Jr. (1998). Microbial complexes in subgingival plaque. J. Clin. Periodontol. 25, 134–144. 10.1111/j.1600-051X.1998.tb02419.x 9495612

[B243] Socransky S. S. Haffajee A. D. Smith G. L. Dzink J. L. (1987). Difficulties encountered in the search for the etiologic agents of destructive periodontal diseases. J. Clin. Periodontol. 14, 588–593. 10.1111/j.1600-051X.1987.tb01520.x 3320100

[B244] Socransky S. S. Manganiello S. D. (1971). The oral microbiota of man from birth to senility. J. Periodontol. 42, 485–496. 10.1902/jop.1971.42.8.485 4998039

[B245] Socransky S. S. Smith C. Martin L. Paster B. J. Dewhirst F. E. Levin A. E. (1994). “Checkerboard” DNA-DNA hybridization. BioTechniques 17, 788–792. 7833043

[B246] Sokol C. L. Luster A. D. (2015). The chemokine system in innate immunity. Cold Spring Harb Perspect Biol 7. 10.1101/cshperspect.a016303 PMC4448619 25635046

[B247] Sommer F. Backhed F. (2013). The gut microbiota–masters of host development and physiology. Nat. Rev. Microbiol. 11, 227–238. 10.1038/nrmicro2974 23435359

[B248] Sprent J. (2017). T cell–B cell collaboration. Nat. Rev. Immunol. 17, 532–532. 10.1038/nri.2017.62 28555672

[B249] Sridharan A. Esposo M. Kaushal K. Tay J. Osann K. Agrawal S.. (2011). Age-associated impaired plasmacytoid dendritic cell functions lead to decreased CD4 and CD8 T cell immunity. Age 33, 363–376. 10.1007/s11357-010-9191-3 20953722 PMC3168606

[B250] Stebbins C. E. Galán J. E. (2001). Structural mimicry in bacterial virulence. Nature 412, 701–705. 10.1038/35089000 11507631

[B251] Stephan R. M. Miller B. F. (1943). A quantitative method for evaluating physical and chemical agents which modify production of acids in bacterial plaques on human teeth. J. Dent. Res. 22, 45–51. 10.1177/00220345430220010601

[B252] Strohmeier G. R. Fenton M. J. (1999). Roles of lipoarabinomannan in the pathogenesis of tuberculosis. Microbes Infect. 1, 709–717. 10.1016/S1286-4579(99)80072-0 10611748

[B253] Sullivan A. Edlund C. Nord C. E. (2001). Effect of antimicrobial agents on the ecological balance of human microflora. Lancet Infect. Dis. 1, 101–114. 10.1016/S1473-3099(01)00066-4 11871461

[B254] Sun L. Zhang X. Zhang Y. Zheng K. Xiang Q. Chen N.. (2019). Antibiotic-induced disruption of gut microbiota alters local metabolomes and immune responses. Front. Cell. Infect. Microbiol. 9:99. 10.3389/fcimb.2019.00099 31069173 PMC6491449

[B255] Syndergaard B. Al-Sabbagh M. Kryscio R. J. Xi J. Ding X. Ebersole J. L.. (2014). Salivary biomarkers associated with gingivitis and response to therapy. J. Periodontol. 85, e295–e303. 10.1902/jop.2014.130696 24502627 PMC4390171

[B256] Takahashi N. Nyvad B. (2011). The role of bacteria in the caries process: ecological perspectives. J. Dent. Res. 90, 294–303. 10.1177/0022034510379602 20924061

[B257] Takahashi N. Nyvad B. (2016). Ecological hypothesis of dentin and root caries. Caries Res. 50, 422–431. 10.1159/000447309 27458979

[B258] Takeuchi O. Hoshino K. Akira S. (2000). Cutting edge: TLR2-deficient and MyD88-deficient mice are highly susceptible to *Staphylococcus aureus* infection. J. Immunol. 165, 5392–5396. 10.4049/jimmunol.165.10.5392 11067888

[B259] Takeuchi O. Sato S. Horiuchi T. Hoshino K. Takeda K. Dong Z.. (2002). Cutting edge: role of Toll-like receptor 1 in mediating immune response to microbial lipoproteins. J. Immunol. 169, 10–14. 10.4049/jimmunol.169.1.10 12077222

[B260] Teng T. S. Ji A. L. Ji X. Y. Li Y. Z. (2017). Neutrophils and immunity: from bactericidal action to being conquered. J. Immunol. Res. 2017:9671604. 10.1155/2017/9671604 28299345 PMC5337389

[B261] Terashima A. Takayanagi H. (2018). Overview of osteoimmunology. Calcif. Tissue Int. 102, 503–511. 10.1007/s00223-018-0417-1 29589061

[B262] Tordesillas L. Berin M. C. (2018). Mechanisms of oral tolerance. Clin. Rev. Allergy Immunol. 55, 107–117. 10.1007/s12016-018-8680-5 29488131 PMC6110983

[B263] Trautmann S. Barghash A. Fecher-Trost C. Schalkowsky P. Hannig C. Kirsch J.. (2019). Proteomic analysis of the initial oral pellicle in caries-active and caries-free individuals. Proteomics Clin. Appl. 13:e1800143. 10.1002/prca.201800143 30548171

[B264] Treerat P. Redanz U. Redanz S. Giacaman R. A. Merritt J. Kreth J. (2020). Synergism between *Corynebacterium* and *Streptococcus sanguinis* reveals new interactions between oral commensals. ISME J. 14, 1154–1169. 10.1038/s41396-020-0598-2 32020052 PMC7174362

[B265] Tsiagbe V. K. Fine D. H. (2012). The impact of bacteria-induced adaptive immune responses in periodontal disease. Periodontal Dis. A Clin. Guide 93, 93–106. 10.5772/26831

[B266] Uzel N. G. Teles F. R. Teles R. P. Song X. Q. Torresyap G. Socransky S. S.. (2011). Microbial shifts during dental biofilm re-development in the absence of oral hygiene in periodontal health and disease. J. Clin. Periodontol. 38, 612–620. 10.1111/j.1600-051X.2011.01730.x 21488936 PMC3177321

[B267] Uzozie A. C. Aebersold R. (2018). Advancing translational research and precision medicine with targeted proteomics. J. Proteomics 189, 1–10. 10.1016/j.jprot.2018.02.021 29476807

[B268] van Crevel R. Ottenhoff T. H. Van Der Meer J. W. (2002). Innate immunity to *Mycobacterium tuberculosis*. Clin. Microbiol. Rev. 15, 294–309. 10.1128/CMR.15.2.294-309.2002 11932234 PMC118070

[B269] van der Hoeven J. S. Franken H. C. (1984). Effect of fluoride on growth and acid production by *Streptococcus mutans* in dental plaque. Infect. Immun. 45, 356–359. 10.1128/IAI.45.2.356-359.1984 6746094 PMC263229

[B270] Van Dyke T. E. (2017). Pro-resolving mediators in the regulation of periodontal disease. Mol. Aspects Med. 58, 21–36. 10.1016/j.mam.2017.04.006 28483532 PMC5660638

[B271] Vila T. Rizk A. M. Sultan A. S. Jabra-Rizk M. A. (2019). The power of saliva: antimicrobial and beyond. PLoS Pathog. 15:e1008058. 10.1371/journal.ppat.1008058 31725797 PMC6855406

[B272] Waerhaug J. (1956). Effect of rough surfaces upon gingival tissue. J. Dent. Res. 35, 323–325. 10.1177/00220345560350022601 13319560

[B273] Wang K. Wang Y. Wang X. Ren Q. Han S. Ding L.. (2018). Comparative salivary proteomics analysis of children with and without dental caries using the iTRAQ/MRM approach. J. Transl. Med. 16:11. 10.1186/s12967-018-1388-8 29351798 PMC5775567

[B274] Weng N. P. (2006). Aging of the immune system: how much can the adaptive immune system adapt? Immunity 24, 495–499. 10.1016/j.immuni.2006.05.001 16713964 PMC2266981

[B275] Williams R. C. Barnett A. H. Claffey N. Davis M. Gadsby R. Kellett M.. (2008). The potential impact of periodontal disease on general health: a consensus view. Curr. Med. Res. Opin. 24, 1635–1643. 10.1185/03007990802131215 18452645

[B276] Willing B. P. Russell S. L. Finlay B. B. (2011). Shifting the balance: antibiotic effects on host-microbiota mutualism. Nat. Rev. Microbiol. 9, 233–243. 10.1038/nrmicro2536 21358670

[B277] Willmann M. Vehreschild M. Biehl L. M. Vogel W. Dorfel D. Hamprecht A.. (2019). Distinct impact of antibiotics on the gut microbiome and resistome: a longitudinal multicenter cohort study. BMC Biol. 17:76. 10.1186/s12915-019-0692-y 31533707 PMC6749691

[B278] Xu X. He J. Xue J. Wang Y. Li K. Zhang K.. (2015). Oral cavity contains distinct niches with dynamic microbial communities. Environ. Microbiol. 17, 699–710. 10.1111/1462-2920.12502 24800728

[B279] Yost S. Duran-Pinedo A. E. Teles R. Krishnan K. Frias-Lopez J. (2015). Functional signatures of oral dysbiosis during periodontitis progression revealed by microbial metatranscriptome analysis. Genome Med. 7:27. 10.1186/s13073-015-0231-6 25918553 PMC4410737

[B280] Yu X. Feng B. He P. Shan L. (2017). From chaos to harmony: responses and signaling upon microbial pattern recognition. Annu. Rev. Phytopathol. 55, 109–137. 10.1146/annurev-phyto-080516-035649 28525309 PMC6240913

[B281] Zhang F. Tanaka H. Kawato T. Kitami S. Nakai K. Motohashi M.. (2011). Interleukin-17A induces cathepsin K and MMP-9 expression in osteoclasts via celecoxib-blocked prostaglandin E2 in osteoblasts. Biochimie 93, 296–305. 10.1016/j.biochi.2010.10.001 20937352

[B282] Zhang S. Yu N. Arce R. M. (2020). Periodontal inflammation: integrating genes and dysbiosis. Periodontol. 2000 82, 129–142. 10.1111/prd.12267 31850627 PMC6924568

[B283] Zhou J. Yao Y. Jiao K. Zhang J. Zheng X. Wu F.. (2017). Relationship between gingival crevicular fluid microbiota and cytokine profile in periodontal host homeostasis. Front. Microbiol. 8:2144. 10.3389/fmicb.2017.02144 29163429 PMC5672786

[B284] Zhou L. N. Bi C. S. Gao L. N. An Y. Chen F. Chen F. M. (2019). Macrophage polarization in human gingival tissue in response to periodontal disease. Oral Dis. 25, 265–273. 10.1111/odi.12983 30285304

